# Reversal of PCNA Ubiquitylation by Ubp10 in *Saccharomyces cerevisiae*

**DOI:** 10.1371/journal.pgen.1002826

**Published:** 2012-07-19

**Authors:** Alfonso Gallego-Sánchez, Sonia Andrés, Francisco Conde, Pedro A. San-Segundo, Avelino Bueno

**Affiliations:** 1 Instituto de Biología Molecular y Celular del Cáncer, Universidad de Salamanca/CSIC, Salamanca, Spain; 2 Instituto de Biología Funcional y Genómica, Universidad de Salamanca/CSIC, Salamanca, Spain; 3 Departamento de Microbiología y Genética, Universidad de Salamanca/CSIC, Salamanca, Spain; University of Washington, United States of America

## Abstract

Regulation of PCNA ubiquitylation plays a key role in the tolerance to DNA damage in eukaryotes. Although the evolutionary conserved mechanism of PCNA ubiquitylation is well understood, the deubiquitylation of ubPCNA remains poorly characterized. Here, we show that the histone H2B^K123^ ubiquitin protease Ubp10 also deubiquitylates ubPCNA in *Saccharomyces cerevisiae*. Our results sustain that Ubp10-dependent deubiquitylation of the sliding clamp PCNA normally takes place during S phase, likely in response to the simple presence of ubPCNA. In agreement with this, we show that Ubp10 forms a complex with PCNA *in vivo*. Interestingly, we also show that deletion of *UBP10* alters in different ways the interaction of PCNA with DNA polymerase ζ–associated protein Rev1 and with accessory subunit Rev7. While deletion of *UBP10* enhances PCNA–Rev1 interaction, it decreases significantly Rev7 binding to the sliding clamp. Finally, we report that Ubp10 counteracts Rad18 E3-ubiquitin ligase activity on PCNA at lysine 164 in such a manner that deregulation of Ubp10 expression causes tolerance impairment and MMS hypersensitivity.

## Author Summary

DNA damage is a major source of genome instability and cancer. A universal mechanism of DNA damage tolerance is based on translesion synthesis (TLS) by specialized low-fidelity DNA polymerases capable of replicating over DNA lesions during replication. Translesion synthesis requires the switch between replicative and TLS DNA polymerases, and this switching is controlled through the ubiquitylation of the proliferating-cell nuclear antigen (PCNA), a processivity factor for DNA synthesis. It is thought that DNA polymerase switching is a reversible process that has a favorable outcome for cells in the prevention of irreversible DNA replication forks collapse. However, the low-fidelity nature of TLS polymerases has unfavorable consequences like the increased risk of mutations opposite to DNA lesions. Here we identify Ubp10 as an enzyme controlling PCNA deubiquitylation in the model yeast *S. cerevisiae*. The identification of Ubp10 is a first step that will allow us to understand its biological significance and its potential role as part of a safeguard mechanism limiting the residence time of TLS DNA polymerases on replicating chromatin in eukaryotes.

## Introduction

In living cells, tolerance mechanisms ensure that DNA can be replicated when it is damaged. These mechanisms prevent irreversible DNA replication fork collapse when the replisome encounters bulky lesions at damaged sites that block progression of replicative DNA polymerases [1], [2]. DNA lesions are bypassed either by a mechanism involving low stringency DNA polymerases called translesion synthesis (TLS) polymerases or by promoting template-switching between nascent chains within the same replication fork [2]–[6]. It is thought that both mechanisms efficiently prevent replisome stalling at damaged sites. The use of TLS polymerases may be mutagenic because they induce an error-prone process that causes damaged-dependent mutations. However, it has been shown that in yeast ultraviolet-radiation-induced DNA lesions are predominantly bypassed via translesion synthesis [7].

Eukaryotes ubiquitylate proliferating-cell nuclear antigen (PCNA) to signal damaged DNA and regulate the choice of alternative pathways to bypass DNA lesions during S-phase, therefore, to tolerate DNA damage [2]–[6]. The sliding clamp PCNA is monoubiquitylated at Lys164 by the Rad6-Rad18 (E2–E3) ubiquitin ligase complex in response to endogenous or exogenous damage causing disruptive covalent modifications of DNA interfering with high-fidelity replicative polymerases during S phase. Mono-ubiquitylated PCNA (ubPCNA) enhances the affinity of error-prone TLS DNA polymerases which facilitate translesion synthesis bypass. Then, the Mms2-Ubc13-Rad5 ubiquitin ligase complex may further ubiquitylate Lys164-mono-ubiquitylated PCNA to promote template switching, the error-free component of the bypass that involves sister-strand pairing [5], [8] and references therein). This regulatory mechanism based on covalent modifications of the Lys164 of the sliding clamp PCNA is a solidly established model conserved in all eukaryotes [2]–[5], [9].

Ubiquitylation of Lys164-PCNA (ubPCNA) greatly enhances binding of the sliding clamp with TLS polymerases [10]. In contrast with replicative enzymes, TLS polymerases are low fidelity DNA polymerases, non-processive enzymes that lack any proofreading activity but capable of replicating over DNA lesions [11] (and references there in). Indeed, TLS polymerases are DNA damage-tolerant enzymes but also mutagenic because they may incorporate mispaired deoxynucleotides opposite to lesions (damaged template) in an error-prone process [12], [13] (and references there in). Because of their low fidelity and low processivity when incorporating deoxynucleotides across from damaged and undamaged base pairs [12], [14]–[17], cells need to keep TLS DNA polymerases from sampling replicative DNA more that strictly required and/or to prevent them from extended interaction with replication forks. Therefore, cells may need a control mechanism to deubiquitylate ubPCNA as soon as TLS DNA polymerases have been able to replicate over the damaged site.

Human Usp1 has been identified as a protease that deubiquitylates mono-ubPCNA [18]. Upon UV-light induced DNA damage, Usp1 is degraded so that PCNA becomes ubiquitylated [18], [19], suggesting that Usp1 deubiquitylates PCNA continuously in the absence of DNA damage [18]. However, accumulation of ubPCNA does not correlate with Usp1 proteolysis when the progression of replication forks is stalled with HU [20], suggesting either a complex regulation of Usp1 activity (or its subcellular localization) when cells are exposed to other DNA damaging agents or the existence of at least one another PCNA deubiquitylating enzyme in mammals acting in response to other DNA damaging agents. Despite the identification of Usp1, little is known about the deubiquitylation of ubPCNA in any other organism.

In *Saccharomyces cerevisiae*, the protease (or proteases) that deubiquitylates ubPCNA remains unknown. Potential candidates in budding yeast are 17 genes that codify for different ubiquitin-specific proteases. Few of them have been extensively studied while others remain poorly characterized [21]–[23]. These genes are named *UBPs* (from *UBP1* to *UBP17*), where *UBP* stands for ubiquitin protease. Among the ubiquitin-specifc proteases characterized, Ubp10/Dot4 is remarkable; this is a deubiquitylating enzyme related to gene-silencing that regulates histone ubH2B deubiquitylation and helps to localise the histone deacetylase Sir2 complex at telomeres, cryptic mating type loci (HML and HMR) and rDNA loci [24], [25]. Here we describe a new role for Ubp10 in deubiquitylating the sliding clamp ubPCNA. We performed a biochemical screening with yeast UBPs single mutants to identify ubiquitin proteases that might play a role in the reversal of PCNA ubiquitylation and found that *UBP10* mutants accumulate ubiquitylated forms of PCNA. Consistent with a direct role in ubPCNA deubiquitylation, we found that catalyticaly active Ubp10 reverts PCNA ubiquitylation.

## Results

### A biochemical screening identifies Ubp10/Dot4 as a potential DUB for PCNA

**Figure 1:**
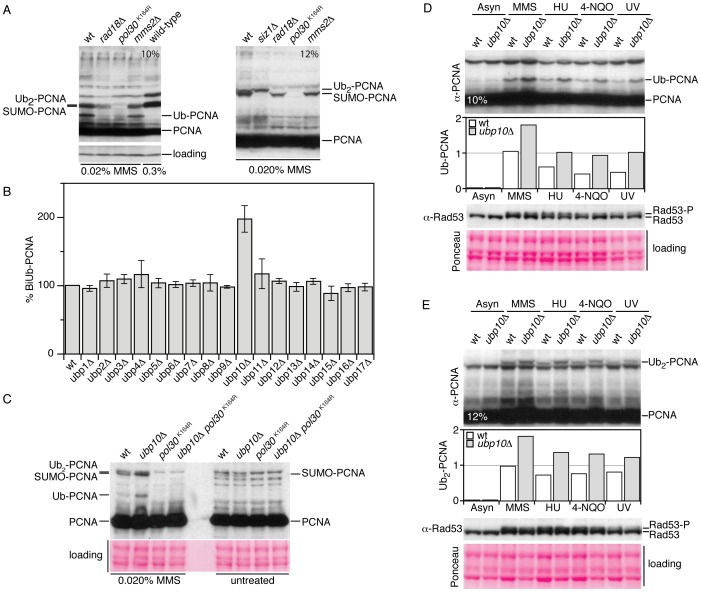
Cells lacking *UBP10* accumulate mono- and di-ubiquitylated PCNA in response to DNA damage and replicative stress. (A) A polyclonal rabbit antibody that specifically detects PCNA forms in yeast cell extracts. Immunoblot analysis with (affinity purified) rabbit α-PCNA antibody of TCA-protein extracts from wild-type, *rad18Δ* (unable to ubiquitylate PCNA), *pol30^K164R^* (unable to ubiquitylate or SUMOylate PCNA), *mms2Δ* (unable to biubiquitylate PCNA) and *siz1Δ* (unable to SUMOylate PCNA) cells treated 90 minutes with 0.020% MMS and resolved in 10% or 12% polyacrylamide gels (as indicated), note that right lane of the 10% gel correspond to wild-type cells treated with 0.3% MMS (conditions where only SUMOylated PCNA forms are detected). (B) Di-ubiquitylated PCNA accumulation in MMS-treated single *ubp1* to *ubp17* deletions in *S.cerevisiae*. Graph of di-ubiquitylated PCNA accumulation in 0.020% MMS-treated single *UBP1-17* deletions in *S.cerevisiae*. Wild-type and single mutant cells exponentially grown at 30°C were treated 60 minutes with 0.020% MMS. TCA-cell extracts were analyzed for PCNA ubiquitylation by Western blot, quantitated and plotted. Average values from three independent assays are plotted. (C) Immunodetection of ubiquitylated forms of PCNA in wild-type, *ubp10Δ*, *pol30^K164R^* and *ubp10Δ pol30^K164R^* TCA-cell extracts to show that *UBP10* mutant cells accumulate K164 mono-ub and di-ubPCNA forms. Immunodetection of mono-ubiquitylated (D) and di-ubiquitylated PCNA (E) in wild-type and *ubp10Δ* cells treated with 0.020% MMS, 200 mM HU, 0.2 µg/ml 4-NQO and 100 J/m^2^ UV-light (as indicated). Rad53 phosphorylation was used to test checkpoint activation upon treatments.

In yeast, the ubiquitylation of PCNA might be a reversible process catalyzed by deubiquitylating enzymes (or DUBs). Sequence and functional analyses have revealed that in budding yeast there are 17 genes (from *UBP1* to *UBP17*) encoding different ubiquitin-specific processing proteases and thus potential candidates to deubiquitylate PCNA. To identify ubiquitin proteases that might play a role in the reversal of PCNA ubiquitylation, we examined PCNA ubiquitylation patterns of *Saccharomyces cerevisiae* strains lacking individual ubiquitin proteases. To detect modified forms of this sliding clamp we used a polyclonal rabbit antibody that specifically detects PCNA in *S.cerevisiae* cell extracts (Figure 1A). As shown in Figure 1B, *ubp10Δ* mutant cells accumulated di-ubiquitylated PCNA forms, a phenotype consistent with defects in deubiquitylation of this sliding clamp. This phenotype (the accumulation of ubiquitylated PCNA) was also observed in cells expressing a version of Ubp10 that lacks catalytic activity (*ubp10* ^C371S^) (see later), a catalytic inactive form previously described [25]. We also found that the ubiquitylated PCNA forms accumulated in *ubp10Δ* mutant cells were covalent modifications on Lysine 164 of the sliding clamp (Figure 1C and Figure S1).

Ubp10 and Ubp8 are the ubiquitin proteases that remove monoubiquitin from histone H2B [24], [25]. Although these H2B-deubiquitylating enzymes have distintc functions [26], deletion of both *UBP8* and *UBP10* results in a synergistic increase in H2B ubiquitylation levels suggesting that they regulate the global balance of that histone modification [24], [25]. Thus, even though we detected normal levels of PCNA modifications in *ubp8Δ* mutant cells, we tested whether or not deletion of *UBP8* in a *ubp10Δ* mutant further increased PCNA ubiquitylation levels. We found that the accumulation of ubPCNA was specific to *ubp10Δ* (Figure S2).

### Cells lacking *UBP10* accumulate mono- and di-ubiquitylated PCNA in response to DNA damage and replicative stress

It has been shown that the ubiquitylation of PCNA is restricted to, although separable from, S-phase [7], [27], [28]. Under physiological circumstances active DNA replication forks are required for PCNA ubiquitylation [27]. In fact, PCNA ubiquitylation is induced by chemicals that cause disruptive covalent modifications of DNA, blocking replication and that involve the accumulation of single-stranded DNA. Thus, in *S. cerevisiae*, PCNA is ubiquitylated during S-phase in response to the detection of DNA lesions caused by methyl methane sulfonate (MMS), hydroxyurea (HU), 4-nitroquinoline 1-oxide (4-NQO), UV light, hydrogen peroxide (H_2_O_2_) and ionizing radiation [27]. We therefore wondered whether *ubp10Δ* mutants accumulate more ubiquitylated PCNA than wild-type cells in response to all these types of inducers. As shown for MMS, HU, 4-NQO and UV light (Figure 1D and 1E), we found that *ubp10Δ* mutant cells accumulated increased levels of ubiquitylated PCNA as compared to control wild-type cells. This observation indicates that *in vivo* Ubp10 modulates the level of DNA damaged-induced PCNA ubiquitylation.

### Overproduction of Ubp10 reverts PCNA ubiquitylation and sensitizes cells to MMS–induced DNA damage

**Figure 2:**
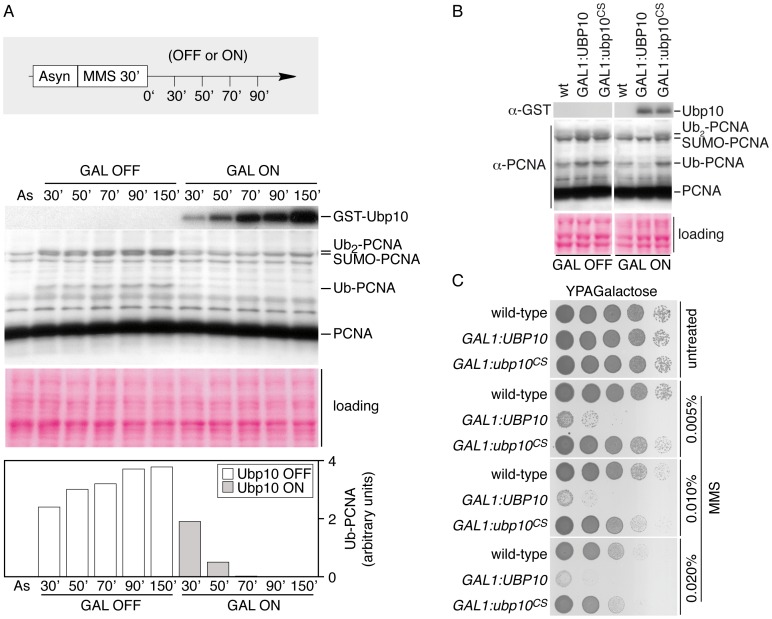
*GAL1*-driven overproduction of *UBP10* reverts PCNA ubiquitylation in response to DNA damage. (A) Time-course analysis of active GST-Ubp10 induction. An asynchronously growing culture of *GAL1,10:GST-UBP10*, incubated in raffinose as unique carbon source, was incubated 30 minutes in the presence of 0.02% MMS. Expression of GST-Ubp10 was either repressed by adding glucose (GAL OFF) or induced with galactose (GAL ON) in the continuous presence of the alkylating chemical (as described). Samples were taken at indicated intervals and processed for immunodetection of modified PCNA forms, PCNA, GST-Ubp10 and Rad53. Ponceau staining of the blotted protein extracts is shown. Mono-ubiquitylated PCNA was quantitated, normalized and plotted. (B) Catalytically active Ubp10 reverts PCNA ubiquitylation *in vivo*. Immunodetection of ubiquitylated and di-ubiquitylated PCNA forms in wild-type cells and in cells reppressed (GAL OFF) or induced (GAL ON) for GST-Ubp10 or GST-Ubp10^CS^ expression, after a 90 minutes treatment with 0.020% MMS. TCA-obtained cells extracts were processed for immunoblotting with α-PCNA and α-GST antibodies. Ponceau staining of the blotted protein extracts is shown for loading control. (C) Ectopic expression of a catalytically active Ubp10 ubiquitin protease hypersensitizes cells to MMS-induced DNA damage. Ten-fold dilutions of equal numbers of cells of wild-type, *GAL1,10:GST-UBP10* and *GAL1,10:GST-ubp10^C371S^* were incubated at 25°C in the absence or the presence of indicated percentages of MMS for 72 hours and photographed.

The increased levels of PCNA ubiquitylation observed in *UBP10* mutant cells suggested that Ubp10 could be a potential candidate for the deubiquitylation of PCNA *in vivo*. We therefore analyzed the ability of Ubp10 to counteract MMS-induced ubiquitylation of PCNA when overproduced. We examined PCNA ubiquitylation in strains in which expression of *UBP10* was regulated by the strong galactose-inducible *GAL1,10* promoter. Exponentially growing cultures were treated with MMS. Then, the expression of *UBP10* was either induced or repressed by adding galactose or glucose, respectively. Samples were taken at regular intervals and processed for Western analysis of PCNA ubiquitylation (Figure 2A). Overexpression of *UBP10* resulted in rapid reversion of PCNA ubiquitylation, consistent with a role as an ubiquitin-specific processing protease for PCNA. Interestingly, both mono- and di-ubiquitylated PCNA forms rapidly disappeared in cells overexpressing *UBP10*, suggesting that Ubp10 also deubiquitylates di-ubPCNA forms. These deubiquitylation events depended on the protease activity of Ubp10 as a catalytically inactive Ubp10^C371S^ mutant form was unable to deubiquitylate PCNA *in vivo* in similar conditions (Figure 2B). We have also observed that Ubp10 overproduction reverts ubiquitylation of PCNA induced by treatments with HU, 4-NQO and UV radiation. In summary, these experiments indicate that overexpression of catalytically active Ubp10 can deubiquitylate ubPCNA *in vivo*. Importantly, this *in vivo* reaction did not require any other *UBP* gene, as active Ubp10 did deubiquitylate ubPCNA in any single *UBP1-17* deletion (Figure S3).

Yeast PCNA mutants lacking the ubiquitin/SUMO-conjugation site K164 or mutated in the PCNA^K164^-E3 ubiquitin ligase Rad18 are hypersensitive to MMS (and other DNA damaging agents) because the ubiquitylation of this K164 amino acid residue is critical to tolerate DNA damage [29]. It is then reasonable to predict that the overexpression of the K164-ubPCNA ubiquitin-specific protease will counteract Rad18 activity and induce MMS hypersensitivity. Therefore, we exposed *UBP10*-overexpressing cells to the chronic presence of the alkylating chemical and found, as predicted, that high levels of expression of the catalytically active form of this ubiquitin protease (but not the inactive Ubp10^C371S^ form) induced hypersensitivity to MMS (Figure 2C). Significantly, this effect was specifically related to high levels of expression of Ubp10 because overexpression of any other *UBP* gene neither sensitize cells to MMS nor induce PCNA deubiquitylation *in vivo* (Figure S4). Regarding *UBP10* overexpression, two additional and testable predictions can be made, first, the hypersensitivity to MMS should depend on the PCNA lysine 164 modification. To test this prediction we used a simple epistasis analysis to determine the order of function of the *POL30* and *GAL1*,*10*: *UBP10* (Figure S5). We have indeed found that *POL30* is epistatic to *GAL1*,*10*: *UBP10* indicating that the MMS-sensitivity of Ubp10 overproduction depends on the PCNA lysine 164 modification. Second, given that mono-ubiquitylation of the K164 residue of PCNA is in principle important to enhance its interaction with mutagenic TLS polymerases, it is plausible to predict that the mutagenesis frequency of cells overexpressing *UBP10* should be reduced as compared to wild-type cells. We have found that this is the case (Figure S6).

### Catalytically active Ubp10 deubiquitylates PCNA *in vivo* independently from histone H2B deubiquitylation

The above observations correlated the enzymatic activity of Ubp10 with PCNA deubiquitylation *in vivo*. However, these effects may depend on deubiquitylation of histone H2B, as Ubp10 deubiquitylates K123 ubH2B [24], [25]. In order to understand whether deubiquitylation of H2B and PCNA were independent from each other, we repeated our overexpression analysis in a *bre1Δ* mutant background. Bre1 is the E3 ubiquitin ligase that ubiquitylates histone H2B in yeast cells, thus, deletion of the *BRE1* gene impedes H2B^K123^ ubiquitylation [30], [31]. Importantly, *BRE1* deleted cells are viable, providing a tool to answer the question. As shown in Figure S7, overproduction of catalytically active Ubp10 reverts PCNA ubiquitylation and hypersensitize cells to MMS similarly in wild-type and *bre1* mutant cells. These results indicate that Ubp10-dependent PCNA deubiquitylation is functionally separable from ubiquitylation of histone H2B.

### Ubp10 is required for rapid PCNA deubiquitylation after MMS–induced DNA damage

MMS modifies guanines and adenines to methyl derivatives causing DNA base mispairing, inducing DNA damage and slowing down progression of DNA replication forks during S-phase [32]–[35]. MMS also induces ubiquitylation of PCNA in all model organisms tested to date (reviewed in [4]). To further study the role of Ubp10 in the modulation of PCNA ubiquitylation in yeast, we analyzed by Western blot samples taken at regular intervals from wild-type cells treated for 60 minutes with the alkylating chemical and compare them to samples taken from *UBP10* mutant cells in similar conditions (Figure S8). As observed in the Figure S8, wild-type cells ubiquitylate PCNA after the MMS treatment and then actively deubiquitylate the sliding clamp in such way that 45 minutes after the release from the drug treatment ubiquitylated PCNA was barely detectable. In contrast *UBP10* deleted cells maintained steady state levels of ubiquitylated PCNA throughout the experiment, suggesting that these cells lack the appropriate enzyme involved in the deubiquitylation of the modified clamp.

### PCNA interacts *in vivo* with Ubp10

Having observed that deletion and overexpression phenotypes of Ubp10 were consistent with the hypothesis that this ubiquitin-specific protease deubiquitylates PCNA in yeast, we next addressed whether Ubp10 and PCNA interact *in vivo*, as expected for an enzyme-substrate complex.

**Figure 3:**
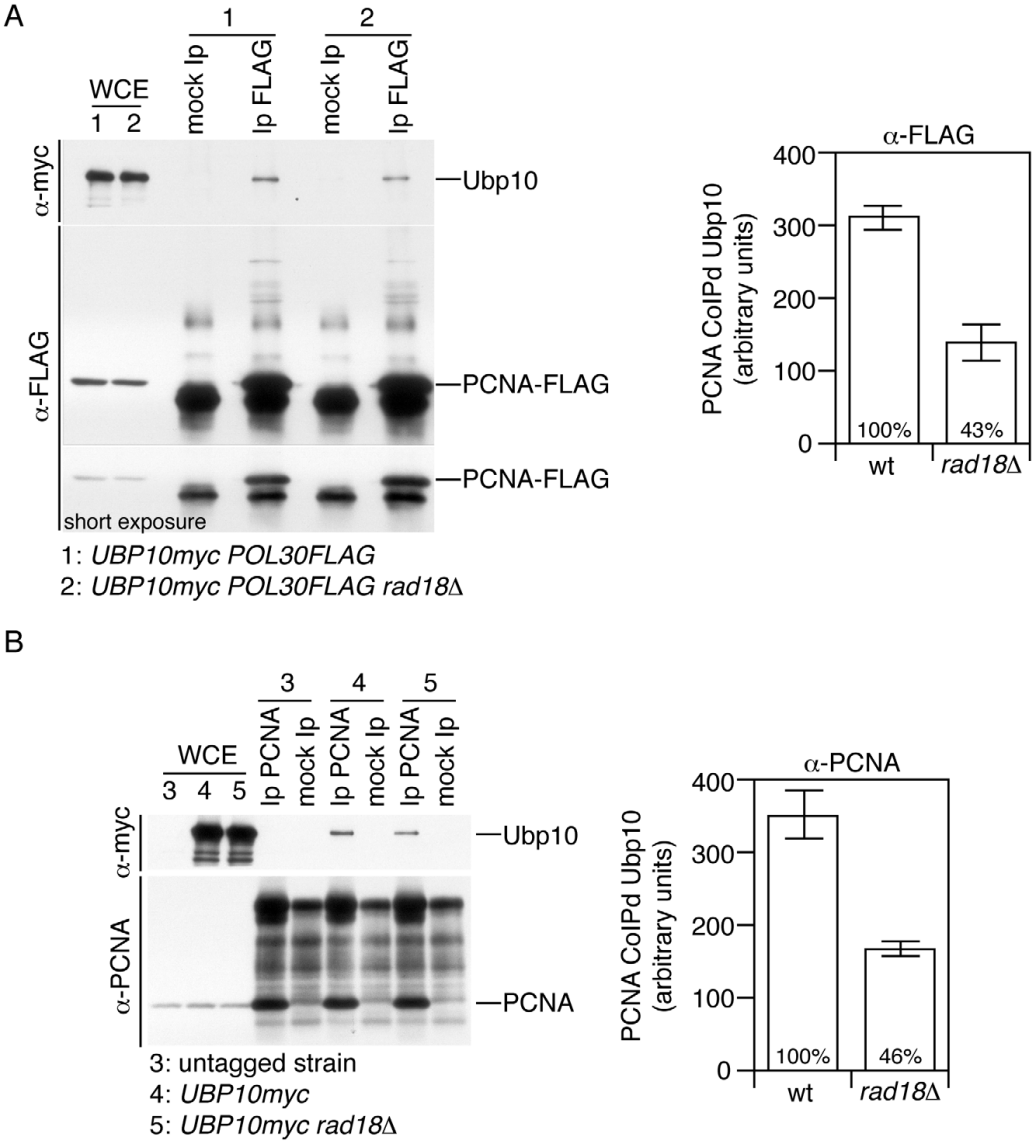
PCNA interacts *in vivo* with Ubp10. (A) The sliding clamp PCNA and Ubp10 specific-ubiquitin protease interact physically *in vivo*. Co-immunoprecipitation assay showing physical interaction between Ubp10-myc and FLAG tagged PCNA. PCNA-FLAG was immunoprecipitated from formaldehyde-crosslinked protein extracts (see methods) both from untreated or 0.020% MMS-treated cells, blots were incubated with α-myc (to detect Ubp10) or α-FLAG (to detect PCNA). The immunoblots shown are those from untreated cells (a similar result was obtained with MMS-treated cells). As indicated the strains used in this assays were *UBP10-myc POL30-FLAG* and *UBP10-myc POL30-FLAG rad18Δ*. Immunoprecipitated Ubp10-myc was quantitated, normalized and plotted. Each immunoprecipitation experiment was repeated three times to gain an estimate of error. (B) Co-immunoprecipitation assay showing physical interaction between Ubp10-myc and PCNA. PCNA was immunoprecipitated both from untreated or 0.020% MMS-treated cells, blots were incubated with α-myc (to detect Ubp10) or α-PCNA. The immunoblots shown are those from MMS-treated cells (a similar result was obtained with untreated cells). As indicated the strains used in this assays were *UBP10-myc* and *UBP10-myc rad18Δ*. Immunoprecipitated Ubp10-myc was quantitated, normalized and plotted. Note that in our experiments we detect Ubp10 interacting with unmodified PCNA (or unmodified PCNA-FLAG).

Addition of single ubiquitin residue to Lys164 of PCNA in yeast is controlled by the E2–E3 complex Rad6–Rad18 during S-phase [7], [28]. Accordingly, the Rad6–Rad18 enzyme complex and its substrate PCNA interact *in vivo*, as has been observed by yeast two-hybrid analyses [29]. We speculated that Ubp10 could form a complex with PCNA in a Rad18 dependent manner, as it has been described previously for other E3-ubiquitin ligases [36]–[38]. If this were true, it could be predicted that these interactions might be detected by co-immunoprecipitation analysis. In particular, we were interested in determining a possible *in vivo* PCNA-Ubp10 interaction at endogenous levels of both proteins. Since we used a C-terminally myc-tagged Ubp10 strain we carefully checked growth rate, gene expression levels, PCNA and histone H2B deubiquitylation and found no differences with untagged wild-type controls, as shown for PCNA (Figure S9). By Western and co-immunoprecipitation assays, we found that Ubp10-myc is stable upon exposure to DNA damage and that Ubp10 binds PCNA throughout the cell cycle and in response to MMS-induced DNA damaged (Figure S9). We then studied Ubp10-PCNA interaction in wild-type and *rad18Δ* mutant cells and observed that Ubp10 and PCNA interact *in vivo* in a Rad18 semi-dependent manner (Figure 3A and 3B). We next tested Ubp10 and Rad18 interaction and found that Rad18 can associate *in vivo* with Ubp10 both in undamaged and exogenously DNA-damaged cells (Figure S10). These results suggest that in yeast cells Ubp10, PCNA and Rad18 could form a complex. These findings, particularly those related to PCNA and Ubp10 interaction, strongly support the hypothesis that Ubp10 is an ubiquitin-specific protease that deubiquitylates PCNA in yeast cells.

### Deletion of *UBP10* results in a net increase in the interaction of Rev1 with PCNA

**Figure 4:**
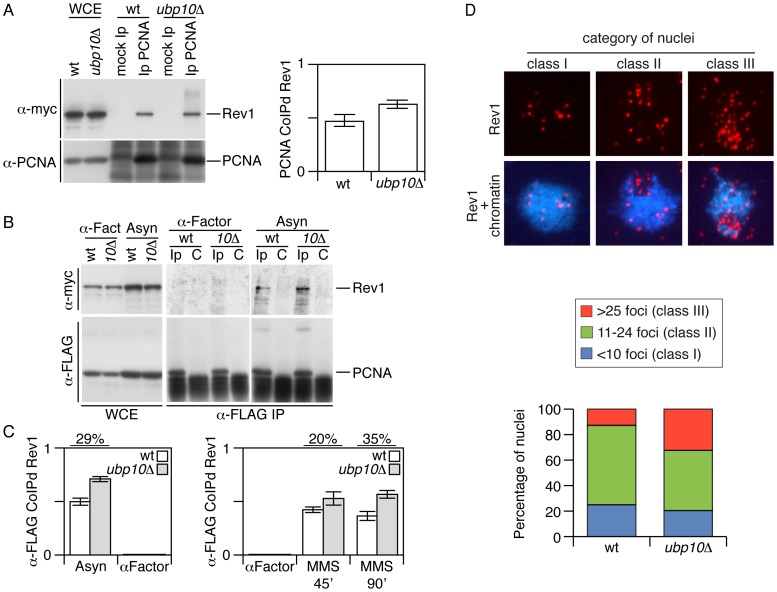
Increased Rev1–PCNA interaction in cells deleted for *UBP10*. (A) Co-immunoprecipitation assay showing physical interaction between Rev1-myc and PCNA. PCNA was immunoprecipitated from 0.020% MMS-treated cells, blots were incubated with α-myc (to detect Rev1) or α-PCNA. The immunoblots shown are those from MMS-treated cells (a comparable result was obtained with untreated cells). As indicated the strains used in this assays were *REV1-myc* and *REV1-myc ubp10Δ*. Immunoprecipitated Rev1-myc was quantitated, normalized (to immunoprecipitated PCNA) and plotted. In (A) as well as in (C), the average and standard deviation values obtained from three independent experiments are plotted. (B) Co-immunoprecipitation assay showing physical interaction between Rev1-myc and PCNA-FLAG. PCNA-FLAG was immunoprecipitated (from protein samples crosslinked with formaldehyde, see methods) from asynchronously growing or α-factor blocked cells (as indicated), blots were incubated with α-myc (to detect Rev1) or α-FLAG (to detect PCNA). As indicated the strains used in this assays were *REV1-myc POL30-FLAG* and *REV1-myc POL30-FLAG ubp10Δ*. (C) Plots of PCNA-FLAG-co-immunoprecipitated Rev1-myc from untreated and 0.02% MMS-treated cells. Rev1-myc samples were quantitated and normalized to immunoprecipitated PCNA-FLAG. Quantitation is shown in bar diagrams. (D) Increased number of chromatin-associated Rev1 foci in MMS-treated *UBP10* mutant yeast cells. Spread nuclei of wild-type and *ubp10Δ* strains carrying *REV1-myc* tagged were stained with DAPI (blue) and anti-myc antibodies (red). Cells were treated with 0.03% MMS for 1 h. The nuclei were classified in three categories according to the number of Rev1 foci. Representative *ubp10Δ* spread nuclei of each class and quantitation of wild-type and *ubp10Δ* nuclei are shown. 47 nuclei were scored for each strain.

In *S.cerevisiae*, *REV1* encodes a deoxycytidyltransferase required for the bypass of abasic sites in damaged DNA. Rev1p forms a complex with the subunits of DNA polymerase ζ Rev3 and Rev7, which are involved in error-prone lesion bypass as yeast TLS DNA polymerases [14], [39]. Furthermore, it has been shown that yeast Rev1 interacts with, and its activity is stimulated by, PCNA [40], [41]. Therefore, we reasoned that the accumulation of mono-ubiquitylated PCNA observed in *UBP10* mutant cells could lead to an increased interaction between PCNA and TLS DNA polymerases, including the TLS-interacting Rev1 protein. We tested this possibility by co-immunoprecipitation assays *in vivo* using strains carrying myc-tagged Rev1 and either wild-type or C-terminal FLAG-tagged PCNA. We detected the reported interaction between the sliding clamp PCNA and the deoxycytidyltransferase Rev1 in the wild-type strain and, importantly, it was increased in cells lacking a functional Ubp10, as predicted (Figure 4A and 4B). We also found that this increase observed in *ubp10Δ* mutant cells was dependent on the PCNA lysine 164 modification (Figure S11). Interestingly, we found that the sliding clamp co-immunoprecipitated Rev1 from asynchronous or MMS-damaged cell cultures (Figure 4C and Figure S12). If this enhacement (in Rev1-PCNA interaction) observed in *ubp10Δ* mutant cells is due to an increase in the ubiquitylation of PCNA, it would be expectable to detect ubiquitylated PCNA in undamaged cells. To our knowledge, detection of ubPCNA in undamaged budding yeast cells remains elusive. However, by immunoprecipitating the sliding clamp from *POL30*-*FLAG* tagged cells, although weakly, we detected ubiquitylated PCNA in asynchronous cultures of exponentially growing wild-type and *UBP10* mutant cells and indeed found that the mutant accumulated ubiquitylated PCNA (Figure S13). This observation supports the correlation between the increase in ubPCNA and the enhancement of Rev1-PCNA interaction in undamaged cells. Finally, we did not observe PCNA-Rev1 interaction in G1 synchronized cells, even though the Rev1 protein was present in the cell extracts (Figure 4B, 4C and Figure S12).

We next analyzed chromatin-associated Rev1 foci and found that, in agreement with the co-immunoprecipitation results, *ubp10*Δ mutant cells had increased numbers of Rev1 foci (mean±s.d.: wild type, 16.64±8.42; *ubp10Δ*, 20.47±10.24). Remarkably, a detailed analysis revealed a significant increment in nuclei with high numbers of Rev1 foci in *UBP10* mutant cells (Figure 4D). In theory, the observed increased interaction between PCNA and Rev1 in *UBP10* deleted cells could be suggestive of a greater TLS activity on replicating chromatin that would result in increased mutagenic rate. Therefore, we next monitored the forward mutation rate to canavanine resistance [42] in undamaged or MMS-damaged *ubp10*Δ mutant cells. However, we found no statistically-significant differences in the mutagenic rate when compared to that of wild-type cells (Figure S14), indicating that increasing levels of PCNA ubiquitylation has no observable impact in the frequency of mutation.

### Analysis of the interaction of Rev3 and Rev7 with PCNA in cells deleted for *UBP10*

**Figure 5:**
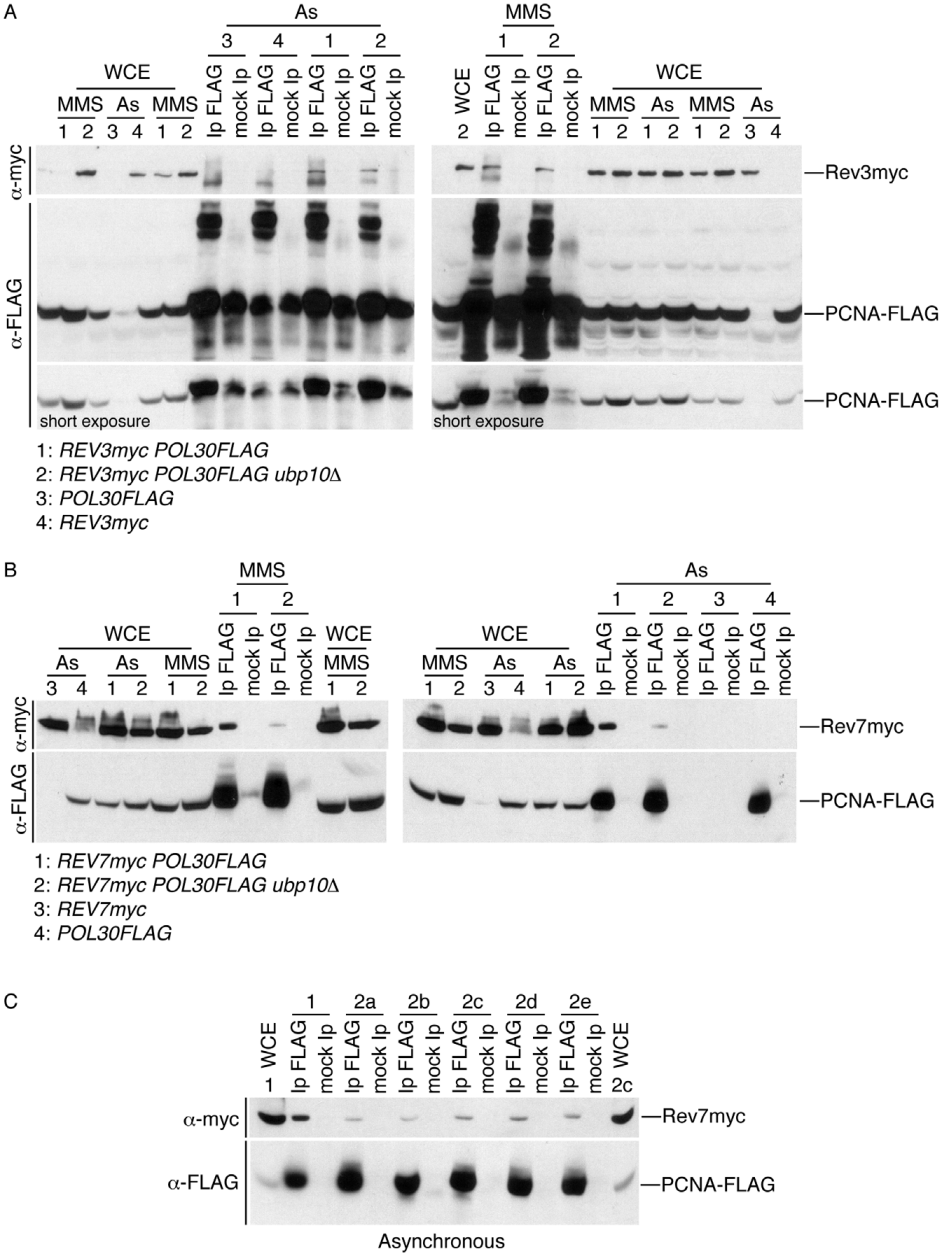
Analysis of Rev3-PCNA and Rev7-PCNA interactions in cells deleted for *UBP10*. (A) Rev3 (DNA polymerase ζ catalytic subunit) interacts with PCNA similarly in wild-type and *ubp10Δ* cells. Co-immunoprecipitation assay showing physical interaction between Rev3-myc and PCNA-FLAG. PCNA-FLAG was immunoprecipitated from asynchronously growing or 0.02% MMS-treated cells (as indicated), blots were incubated with α-myc (to detect Rev3) or α-FLAG (to detect PCNA). As indicated the strains used in this assays were *REV3-myc POL30-FLAG*, *REV3-myc POL30-FLAG ubp10Δ* and single tagged *POL30-FLAG* or *REV3-myc* controls. Whole cell extracts (WCE) and mock Ip controls are also shown as indicated. (B) The interaction of PCNA with Rev7 (an accessory subunit of DNA polymerase ζ) is reduced in cells deleted for *UBP10*. Co-immunoprecipitation assay of Rev7-myc and PCNA-FLAG. PCNA-FLAG was immunoprecipitated from asynchronously growing or 0.02% MMS-treated cells (as indicated), blots were incubated with α-myc (to detect Rev7) or α-FLAG (to detect PCNA). As indicated, the key strains used in this assays were *REV7-myc POL30-FLAG* and *REV7-myc POL30-FLAG ubp10Δ.* Appropriate single tagged, input (WCE) and mock Ip controls are shown. (C) Deletion of *UBP10* alters the interaction of PCNA with Rev7. To assure that deletion of *UBP10* reduced significantly Rev7-PCNA interaction, *UBP10* was deleted in the *REV7-myc POL30-FLAG* strain used in B. Five different *REV7-myc POL30-FLAG ubp10Δ* deletion strains and a *REV7-myc POL30-FLAG* control were used in the co-immunoprecipitation analysis. PCNA-FLAG was immunoprecipitated from asynchronously growing cells, blots were incubated with α-myc (to detect Rev7) or α-FLAG (to detect PCNA). The strains used in this assays were either *REV7-myc POL30-FLAG (1)* or *REV7-myc POL30-FLAG ubp10Δ* (2a, 2b, 2c, 2d and 2e). Input whole cell extracts (WCE) and mock Ip controls are shown. Note that similar amounts of Rev7 are present in whole cell extracts of *REV7-myc POL30-FLAG* (1) and *REV7-myc POL30-FLAG ubp10Δ* (2c) cells.

The Rev1-Rev3/Rev7 complex formation has been succesfully tested in yeast [43], [44]. However, having shown that mutation of *UBP10* enhances Rev1 interaction with PCNA but does not increase mutation frequency (and in order to explain this discrepancy), we wondered whether the Rev3/Rev7 (DNA polymerase ζ) interaction with PCNA was regulated in a different way than the observed for Rev1 in *ubp10Δ* yeast mutant cells. In order to test this hypothesis, we first analysed Rev3-PCNA interaction in wild-type and *ubp10Δ* cells (Figure 5A). By co-immunoprecipitation assays, we found that Rev3, the catalytic subunit of pol zeta, interacts with PCNA in wild-type and *ubp10Δ* mutant strains. We also observed that the amount of Rev3 co-immunoprecipitated with PCNA was similar in both strains either in asynchronous cultures or when cells were treated with MMS. We nex studied the interaction of PCNA with the accessory subunit of DNA polymerase ζ Rev7 (Figure 5B and 5C). Rev7 stimulates the activity of Rev3 [14] and is required for mutagenesis induced after DNA damage in such a manner that deletion of *REV7* decreases mutagenesis frequency in yeast [45]. Significantly, in our co-immunoprecipitation assays we did observe that the interaction of PCNA with Rev7 was greatly reduced in cells deleted for *UBP10* supporting an explanation for the wild-type-like mutagenesis frequency observed in them.

### Cells lacking Ubp10 accumulate mono- and di-ubiquitylated forms of PCNA in response to HU-induced DNA replication blocks

**Figure 6:**
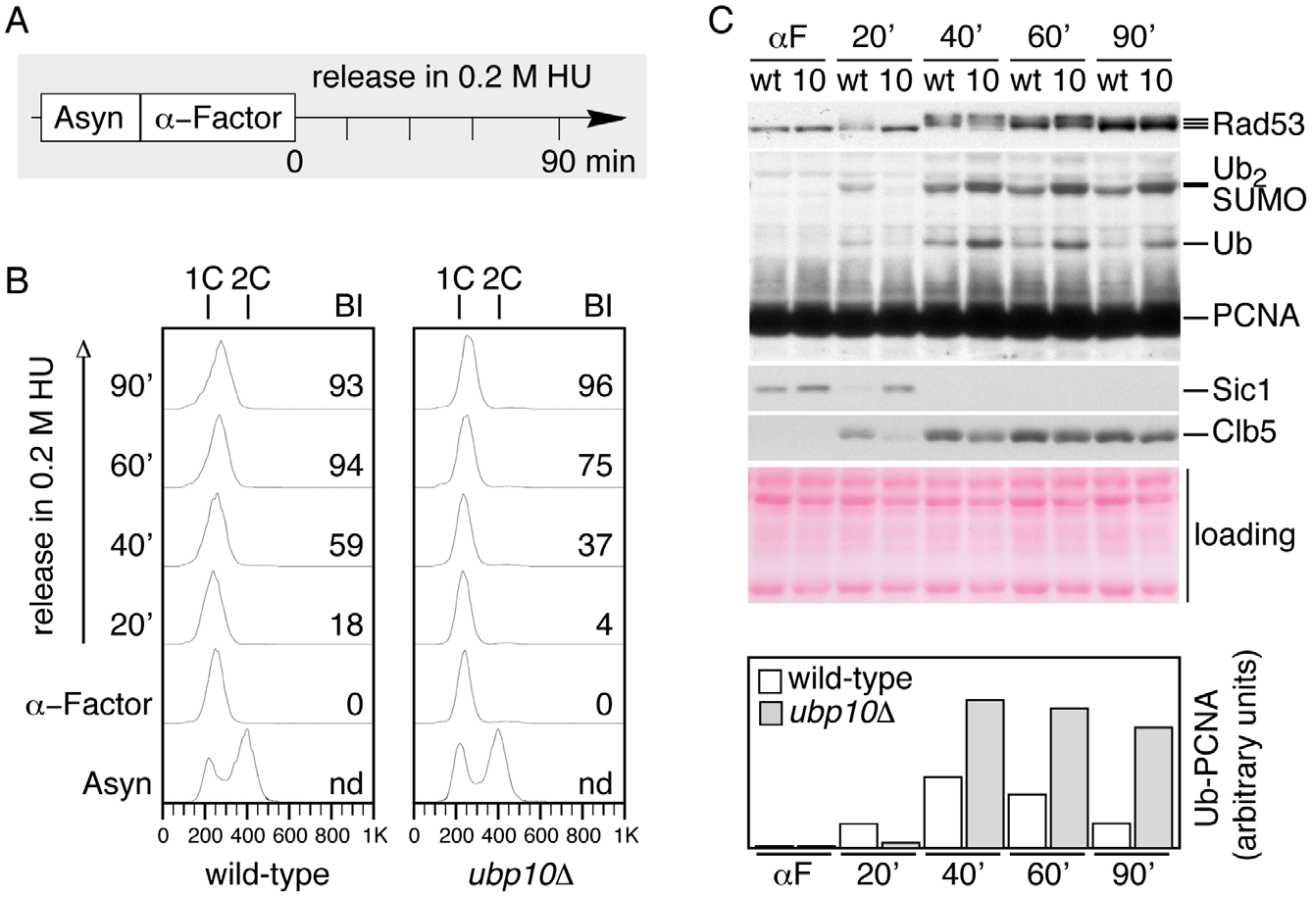
Cells lacking Ubp10 accumulate ubiquitylated PCNA forms early during S-phase in response to HU-induced DNA replication blocks. (A) Experimental design, exponentially growing cultures of wild-type and *ubp10Δ* strains were synchronized with α-factor and then released in 0.2 M HU. Samples were taken at indicated intervals and processed for FACS and Western analysis. (B) FACS analysis showing the checkpoint-induced S phase arrest of asynchronous wild-type and *ubp10Δ* cells during the HU treatment. BI: budding index. (C) Western blot analysis of PCNA, Rad53, Sic1 and Clb5 protein levels in wild-type and *ubp10Δ* cells treated with 0.2 M HU (labeled as wt and 10, respectively). ubPCNA signals were quantitated and normalized to loading controls. Quantitation is shown in bar diagrams. Whereas *ubp10Δ* cells accumulate ubPCNA forms in response to HU, ubPCNA levels declined after the 40 minutes peak in wild-type cells.

**Figure 7:**
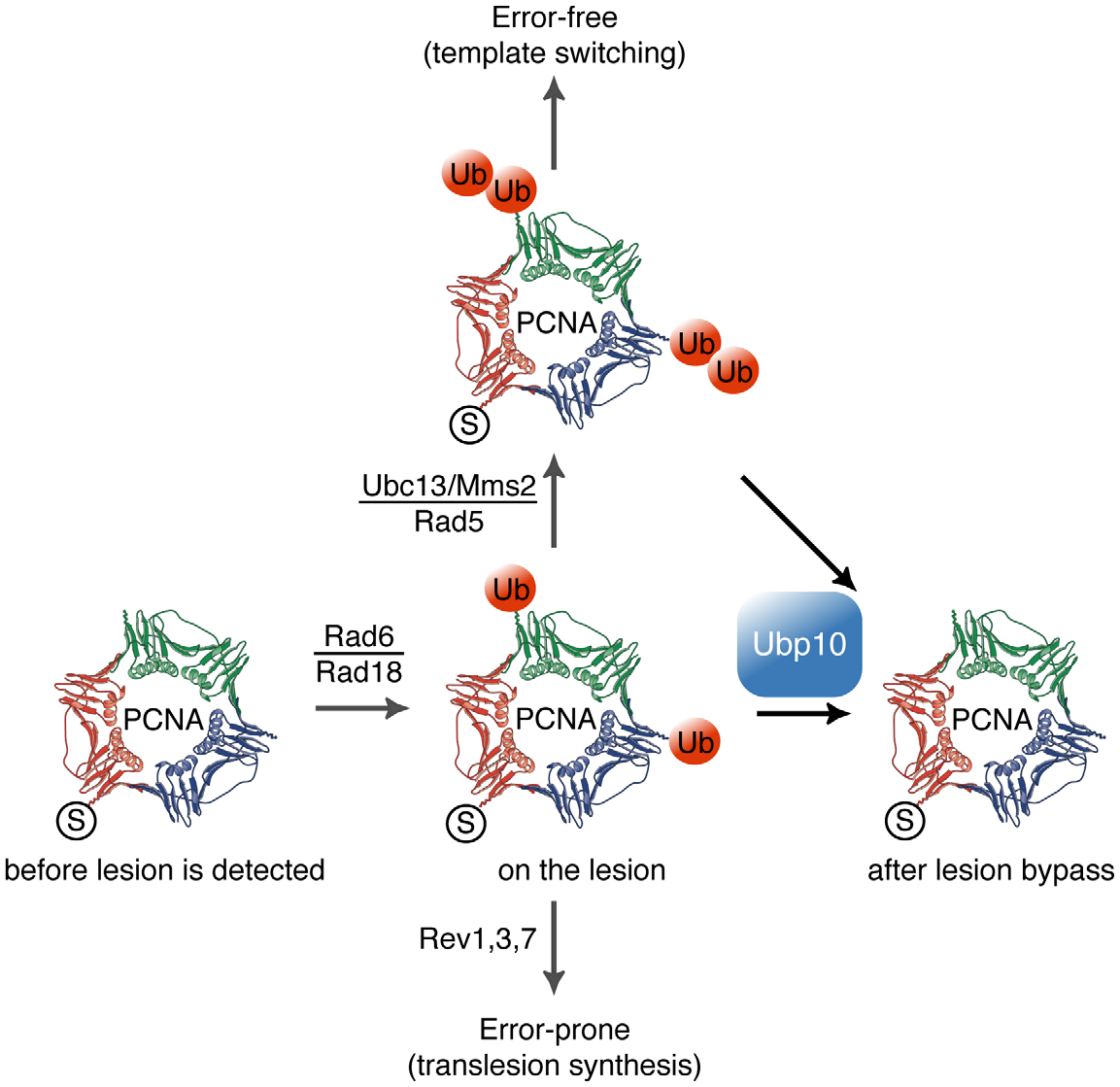
Model for Ubp10 role on the modulation of PCNA ubiquitylation in *S. cerevisiae* cells. SUMOylated PCNA progress with the replisome at replication forks. Detection of bulky lesions on DNA impedes fork progression and induces Rad6/Rad18 ubiquitylation of PCNA; therefore, it enhances ubPCNA-TLS DNA polymerases interaction or further ubPCNA polyubiquitylation (by the Ubc13/Mms2/Rad5 ubiquitin ligase). After lesion bypass, Ubp10 deubiquitylates ubPCNA to allow remodelling of the replisome by switching back to replicative DNA polymerases, resuming rapid and processive DNA replication fork progression.

The evidence presented up to here indicate that the activity of Ubp10 is required for reverting PCNA ubiquitylation but does not addesss when Ubp10-mediated PCNA deubiquitylation takes place during the cell cycle. Therefore, we were next interested in understanding whether deubiquitylation of PCNA occurs during S-phase. Through the depletion of nucleotides, the drug hydroxyurea (HU), an effective ribonuclease reductase inhibitor, causes an early S-phase arrest in *S.cerevisae* cells [46] and induces ubiquitylation of PCNA [27], thus, providing a way to study the regulation of PCNA ubiquitylation in the presence of stalled DNA replication forks. In this scenario, we compared PCNA ubiquitylation in wild-type and *ubp10Δ* mutant cells (Figure 6). Cells in logarithmic growth at 30°C were synchronyzed with α-factor and then released in 0.2M HU at the same temperature and samples (taken at regular intervals) processed for Western analysis of PCNA. We used as S-phase markers PCNA SUMOylation [29], Rad53 activation [35] and Clb5 accumulation [47]–[50]. As recently described [7], [27], [28], we detected PCNA ubiquitylation as soon as cells entered S-phase, coincident with the appearance of PCNA SUMOylation, Rad53 activation (in response to HU) and Clb5 accumulation (Figure 6C). Under the chronic presence of HU, in wild-type cells PCNA ubiquitylation reached a maximum 40 minutes after the release from the pheromone arrest and then started to decline with stalled DNA replication forks as judged from all markers, including DNA content analysis by FACS. The timing of PCNA ubiquitylation observed here correlates well with the recently described timing of association of Rad18 with replicating chromatin in HU treated cells [27]. The decrease in ubPCNA observed in wild-type cells was somewhat surprising; however, it does indicate that yeast cells down-regulate the modification of the clamp during S-phase. In contrast, cells lacking Ubp10 activity, even though they progressed into S-phase later or more slowly than controls (Figure 6B and 6C), accumulated increased amounts of mono and di-ubiquitylated forms of the clamp that remained high all throughout the synchronous experiment (see bar plot for ubPCNA in Figure 6C). The analysis of *ubp10Δ* mutant cells is consistent with the idea that this ubiquitin-specific protease down-regulates PCNA ubiquitylation during S-phase and suggest that Ubp10 is a major deubiquitylating enzyme for ubPCNA in budding yeast cells (see model in Figure 7).

## Discussion

In this work we present clear evidence indicating that Ubp10 controls PCNA deubiquitylation in *S. cerevisiae*. Ubp10 has a well established role as an ubiquitin-specific protease of ubH2B, a role related to gene-silencing (at telomeres, rDNA and cryptic mating type loci), together with Ubp8, the SAGA-associated ubH2B deubiquitylase involved in gene expression [24], [25]. Thus, in combination Ubp8 and Ubp10 regulate the global balance of ubH2B [24], [25]. In addition to this role, here we present results supporting that Ubp10 is an important ubiquitin-specific protease also in removing ubiquitin from ubPCNA in budding yeast. Our observations that wild-type cells deubiquitylate ubPCNA in response to the alkylating chemical MMS or under the chronic presence of HU show that there exists an active control to revert PCNA ubiquitylation in *S.cerevisiae* yeast cells. Moreover, our experiments with *ubp10^C371S^* mutant strains indicate that such control depends on the catalytic activity of Ubp10/Dot4.

*UBP10* deleted cells or cells carrying a catalytically inactive form of Ubp10 accumulate ubPCNA, a phenotype consistent with the idea that *in vivo* Ubp10 is the protease that removes ubiquitin from ubiquitylated PCNA. In agreement with this role, overexpression of active Ubp10 reverts PCNA ubiquitylation and hypersensitizes cells to MMS. Moreover, Ubp10 and the sliding clamp PCNA interact *in vivo* as expected from the formation of and enzyme-substrate complex. Importantly, the function of Ubp10 as ubPCNA ubiquitin-specific protease is separable from histone H2B ubiquitylation, as Ubp10 deubiquitylates ubPCNA in cells lacking Bre1, the E3 ubiquitin ligase that in complex with Rad6 monoubiquitylates histone H2B^K123^ [31], [51]. However, the ubPCNA and ubH2B deubiquitylation roles of Ubp10 might be functionally related. One interesting hypothesis is that Ubp10-dependent deubiquitylation of ubPCNA and ubH2B are inseparable functions. It is arguable that Ubp10 might modulate both replication bypass and histone modification in order lo leave the epigenetic marks unaltered during DNA replication. In fact, it has been inferred from DT40 chicken cells defective in Rev1 that this TLS-associated deoxycytidyl transferase is involved in replication of G4-structured DNA regions and, as a consequence of it, in leaving intact their histone methylation epigenetic marks [52]. Since here we report a functional link between Rev1, PCNA, Rad18 and Ubp10, it is reasonable to surmise that Ubp10 would modulate PCNA ubiquitylation and (the maintenance of) histone imprinting during replication. These modulatory roles are also consistent with the fact that the modulator (Ubp10) might form part of the complexes (PCNA, Rad6-Rad18, Rad6-Bre1) involved in both actions.

An important observation presented in this work is that Ubp10 is able to remove mono-ubiquitin as well as di-ubiquitin from PCNA *in vivo*, suggesting that this ubiquitin protease enzyme may be crucial for keeping TLS polymerases in check as well as for down-regulating the error-free bypass. Thus, a single deubiquitylating enzyme might downregulate both branches of the tolerance pathway to DNA damage in budding yeast.

Where does PCNA deubiquitylation take place? The answer to this simple question is not necessarily trivial, since the localization Ubp10 might be a point of interest for future analysis. Initial studies in formaldehyde-fixed cells suggested that Ubp10 localizes primarily at the nucleus [53]; however, using *in vivo* studies of Ubp10-GFP as well as immunofluorescence analysis of Ubp10-myc on nuclear spreads, we have found that Ubp10 localizes mainly in the rDNA-containing nucleolar region (our own unpublished observations). Thus, does Ubp10 localize permanently to the nucleolus? ChIP evidence has confirmed rDNA loci, telomeres and cryptic mating type loci localization [24], [25], [54] so that Ubp10-dependent deubiquitylation of ubH2B should take place there. Deubiquitylation of ubPCNA may follow a more dynamic pattern (as DNA replication forks move during ongoing replication). Alternatively, and more simply, an undetected fraction of Ubp10 might be permanently located out of the nucleolus or might be released from this nuclear compartment to control the deubiquitylating processes during S-phase and postreplication repair. Future studies will address these alternatives.

As in yeast cells, PCNA ubiquitylation is required for mammalian cell survival after UV irradiation, HU or MMS treatment [55]. In human cells Usp1 deubiquitylates PCNA as well as the Fanconi's anaemia protein FANCD2 [19], [56]–[58]. It has been shown that human Usp1 incessantly deubiquitylates ubPCNA in the absence of DNA damage [18]. Upon UV light-induced DNA damage, Usp1 is (auto)proteolysed, such that PCNA becomes ubiquitylated [18], [19]. Our work has uncovered several differences in the regulation of PCNA deubiquitylation between yeast and human cells. First, we observed that *UBP10* deleted yeast cells accumulate ubiquitylated PCNA forms in response to MMS, HU, UV-light and 4-NQO, suggesting that a single DUB (Ubp10) may control PCNA deubiquitylation in budding yeast. Second, Ubp10 appears to deubiquitylate PCNA during S-phase (when the sliding clamp is modified). Finally, Ubp10 protein levels remained constant when cells are exposed to DNA damage. Thus, it is unlikely that a similar Usp1-like autoregulatory mechanism on yeast Ubp10 ubiquitin protease would exist.

The evidence presented here supported the hypothesis that Ubp10 deubiquitylates PCNA to limit the residence time of TLS polymerases on DNA replication forks during S-phase. We tested this hypothesis directly by studying Rev1-PCNA interaction because Rev1 serves as a scaffold for the polymerase ζ, encoded by *REV3* and *REV7*, for efficient bypass of DNA lesions [59]–[61]. In agreement with this hypothesis, we found that deletion of *UBP10* resulted in an increased interaction between PCNA and Rev1 in undamaged and DNA-damaged cells, and that, in turn, this enhanced interaction resulted in a net increase in Rev1 foci in chromatin. However, in contradiction with an increased number of Rev1 foci, we have also found that deletion of *UBP10* does not increase the mutagenic frequency. A conceivable explanation for this contradiction would be that and additional level of control on TLS polymerases may exist to regulate their activity. In this context, one simple possibility is that DNA polymerase ζ interaction with replicating chromatin may be hindered in *UBP10* deleted cells. Therefore, to explain the observed discrepancy we studied the interaction of DNA polymerase ζ subunits Rev3 and Rev7 with PCNA. Significantly, we have found that DNA polymerase ζ accessory subunit Rev7 requires Ubp10 to fully interact with the sliding clamp PCNA. This observation explains why *ubp10Δ* mutant cells have a wild-type-like mutagenic frequency and, more importantly, it opens the unexpected possibility that Rev1 and DNA polymerase ζ subunits may be regulated in quite distinct ways regarding their interaction with PCNA and, thus, with replicating chromatin. Further studies will be required to test this hypothesis and to study the potencial role of Ubp10 in modulating DNA polymerase ζ subunit Rev7 binding to the sliding clamp PCNA. In summary, our data support that Rev1 interaction with PCNA is modulated by ubiquitylation of PCNA and, thus, follows the classical regulatory model. Here, we propose that Ubp10 participates in this modulation through the deubiquitylation of ubPCNA. However, from the observations presented here we also deduced that Ubp10 may play a direct or indirect role in regulating Rev7 interaction with the sliding clamp apparently in a PCNA ubiquitylation independent manner.

It is proper to mention here that the activity TLS-DNA polymerases activity may be regulated by checkpoint kinases. For example, it has been shown in budding yeast that Rev1 is regulated during the cell cycle [62], and that it is phosphorylated by the Mec1-Ddc2 kinase in response to various types of DNA damages [63]–[65]. Thus, in response to DNA damage, yeast cells would have two different levels of control: first, in modulating the interaction of PCNA and TLS polymerases, and second, in regulating TLS polymerases activity and/or stability. A control mechanism that may be conserved as ATR-mediated phosphorylation of DNA polymerase η is involved in the proper response to UV-mediated DNA damage in human cells [66].

What might be the biological significance of Ubp10-mediated ubPCNA deubiquitylation in budding yeast? It is tempting to say that our results suggest that the biological significance of the control of PCNA deubiquitylation in *S.cerevisiae* is to prevent extended residence time of Rev1 in replicating chromatin. However, there is no unfavorable outcome for yeast cells deleted for *UBP10* as they fail to support a full interaction of (DNA polymerase ζ subunit) Rev7 with PCNA and, consequently, they show a wild-type-like mutagenic frequency. It is true that these opposite effects on Rev1 and Rev7 suggest the hypothesis that Ubp10 has a complex role in modulating TLS subunits interaction with PCNA (and perhaps with replicating chromatin). However, additional studies will be required to test this hypothesis. Significantly, it has been reported the functionality in tolerance of a PCNA mutant form constitutively fused to mono-ubiquitin [67]. Thus, an alternative interpretation of our results is that Ubp10-driven deubiquitylation of ubPCNA may not be that important to tolerate DNA damage in yeast as deletion of *UBP10* has no impact in MMS sensitivity nor leads to a mutator phenotype.

## Materials and Methods

General experimental procedures of yeast Molecular and Cellular Biology were used as described previously [68]–[71].

### Yeast strains, cell culture, and flow cytometry

All the budding yeast used in our studies are listed in Table S1. Yeast strains were grown in rich YPA medium (1% yeast extract, 2% peptone, 50 µg/ml adenine) containing 2% glucose. For block-and–release experiments, cells were grown in YPA with 2% glucose (except where indicated) at 25°C and synchronised with α-factor pheromone in G1 by adding 40 ng/ml (final concentration, 2.5 hours). Cells were then collected by centrifugation and released in fresh media in the absence or in the presence of MMS (or other drugs as indicated). Overexpression experiments with cells grown in YPA medium with 2% raffinose at 25°C were conducted by adding to the medium 2.5% galactose (to induce) or 2% glucose (to repress) and further incubating with/without MMS. For flow cytometry, 10^7^ cells were collected by centrifugation, washed once with water, and fixed in 70% ethanol and processed as described previously [68], [72]. The DNA content of individual cells was measured using a Becton Dickinson FACScan. Cells were prepared for flow cytometry as described [72], [73].

### MMS and drugs sensitivity assays

Exponentially growing or stationary cells were counted and serially diluted in YPA media. Tenfold dilutions of equal numbers of cells were used. 10 µl of each dilution were spotted onto YPAD (2% glucose) or YPAGal (2.5% galactose) plates (always supplemented with 50 µg/ml adenine), YPAD or YPAGal plates containing different concentrations of MMS (Sigma), or HU (Sigma), incubated at 25°C and scanned. MMS plates were always freshly made.

### Mutagenesis assay

Forward mutation analysis at the CAN1 locus was performed essentially as described previously [74]. Cells were grown in rich medium (YPAD or YPAGal) to log phase and MMS (at indicated concentrations) was added to the half of each culture, which were further incubated until the saturation point was reached (24 hours for wild-type, *ubp10Δ* and *ubp10Δ rev3Δ* strains in Figure S14 to 48 hours for wild-type, *GAL1,10:UBP10*, *rev3Δ* and *GAL1,10:UBP10 rev3Δ* strains in Figure S6). Then, cells were plated on solid medium without arginine but containing 60 µg/ml canavanine (Sigma) and also in control YPAD plates (for reference). After 4 days, colonies were counted and the mutagenesis frequency (canavanine resistant cells versus total population) was calculated for each culture. The frequencies provided are mean values of six or more independent cultures of each indicated genotype, in at least three independent experiments.

### Tagging yeast proteins and gene deletion

Tagged alleles were constructed using the single step PCR-based gene modification strategy [75]. A similar strategy was used to generate specific gene deletions. The selection markers used were *KanMX6*, which allows selection with geneticin, *HphMX4*, which allows selection with hygromicin or *NatMX4*, which allows selection with nourseothricin. We used also *LEU2* and *HIS3* markers (as indicated in Table S1). The resulting genomic constructions were confirmed by PCR and sequencing. In the case of tagged alleles, the presence of tagged proteins was confirmed by Western blot.

### Immunoprecipitation, Western blot analysis, and antibodies

#### Protein extract preparation for Western analysis

TCA cell extracts were prepared and analyzed as described previously [70], [76]. SDS-PAGE gels at 15%, 12%, 10% and 7.5% were used for detection of histone H2B, PCNA (12% and 10%) and Rad53, respectively.

#### Protein extract preparation for immunoprecipitations

Soluble protein extracts were prepared basically as described previously [77]. Cells were collected, washed, and broken in HB2T buffer using glass beads. The HB2T buffer contained 60 mM β-glycerophosphate, 15 mM *p*-nitrophenylphosphate, 25 mM 4-morpholinepropanesulfonic acid (pH 7.2), 15 mM MgCl2, 15 mM EGTA, 1 mM dithiothreitol, 0.1 mM sodium orthovanadate, 2% Triton X-100, 1 mM phenylmethylsulfonyl fluoride, and 20 mg/ml leupeptin and aprotinin. The glass beads were washed with 500 ml of HB2T, and the supernatant was recovered. Protein concentrations were measured using the BCA assay kit (Pierce). We repeated the immunopreciptation of PCNA or PCNA-FLAG experiments in the presence of the crosslinking agent formaldehyde (as indicated in Figure legends), and cell extracts were prepared and then processed as for ChIP [69], [78]. After immunoprecipitation of PCNA or PCNA-FLAG, tagged proteins were detected by immunoblotting with specific monoclonal antibodies (the IPs were washed as for ChIP assays, however, it was mixed with Laemmli buffer before incubation at 95°C for 30 min to reverse the crosslinking and denature the eluted proteins). The *in vivo* interactions described in the Results section (in particular PCNA with Ubp10 and PCNA with Rev1) were quantitated from Western analysis of co-immunoprecipitates. In every case, the experiments were repeated three times to gain an estimate of error.

#### Western blotting

Protein extracts and immunoprecipitates were electrophoresed using SDS-polyacrylamide gels ranging from 7.5 to 15%. For Western blots, 40–80 µg of total protein extracts from each sample were blotted onto nitrocellulose, and proteins were detected using a characterized anti-PCNA affinity-purified polyclonal antibody (1∶1500; a generous gift from Dr. Paul Kaufmann). We also used Clb5, Sic1 and Rad53 antibodies from Santa Cruz Biotechnology (used as indicated by the supplier), as well as the 12CA5 monoclonal antibody (Roche Molecular Biochemicals; 1∶500), or the anti-FLAG monoclonal antibody (1∶3000), or the anti-Myc monoclonal antibody (1∶3000). Polyclonal anti-GST antibody (1∶3000) was also used. Horseradish peroxidase-conjugated anti-rabbit, anti-goat, or anti-mouse antibodies (as required) and the ECL kit (GE Healthcare) were used. The antibodies required for immunoblots were used at the indicated dilutions for Western analysis.

### Imaging of cells and citology

Immunofluorescence of nuclear spreads was performed essentially as described previously [71], [79]. The anti-myc tag antibody (clone 4A6, 05-724; Millipore) was used at 1∶500 dilution and the Alexa Fluor 594-conjugated anti-mouse secondary antibody (A11032; Molecular Probes) was used at 1∶200 dilution. Images were captured using a Nikon Eclipse 90i fluorescence microscope equipped with an Orca-AG (Hamamatsu) CCD camera and a PlanApo VC 100×/1.4 objective. Images were processed and analyzed with the MetaMorph software (Molecular Devices). Quantification of chromosome-associated Rev1 was performed by counting the number of Rev1-myc foci in the DAPI-stained area.

## Supporting Information

Figure S1 Immunodetection of ubiquitylated forms of PCNA in yeast TCA-cell extracts to show that *UBP10* mutant cells accumulate K164 but not K127 modified PCNA forms. Immunoblot analysis with α-PCNA antibody of TCA-protein extracts from *pol30^K164R^* (unable to ubiquitylate or SUMOylate PCNA at K164), wild-type (wt), *pol30^K127R^* (unable to ubiquitylate or SUMOylate PCNA at K127), *ubp10Δ*, *ubp10Δ pol30^K127R^*, *ubp10Δ*, G1 wild-type (wt α-factor), *siz1Δ* (unable to SUMOylate PCNA), *mms2Δ* (unable to di-ubiquitylate PCNA), *rad18Δ* (unable to ubiquitylate PCNA), and *ubp10Δ pol30^K164R^* cells treated 90 minutes with 0.020% MMS and resolved in a 12% polyacrylamide gel, note the presence of a sample from untreated wild-type cells (8th lane).
(JPG)

Figure S2 *ubp10* but not *ubp8* mutant cells accumulate ubiquitylated forms of PCNA in response to MMS-induced DNA damage. Immunodetection of mono-ubiquitylated (ubPCNA) and di-ubiquitylated PCNA (Ub_2_-PCNA) in wild-type, *ubp8Δ, ubp10Δ* and *ubp8Δ ubp10Δ* cells treated with 0.020% MMS (as indicated). Ubiquiylated PCNA (ubPCNA) samples were quantified, normalized to loading controls and plotted. Rad53 phosphorylation is used for testing checkpoint activation upon MMS-treatment.
(JPG)

Figure S3 *GAL1*-driven overproduction of *UBP10* reverts PCNA ubiquitylation in any *UBP1-17* deletion. Catalytically active Ubp10 reverts PCNA ubiquitylation *in vivo* in *ubp1Δ* (*1Δ*) to *ubp17Δ* (*17Δ*) single mutants. Immunodetection of K164-monoubiquitylated PCNA forms in wild-type cells (wt), *GAL1*-regulated overexpressing *UBP10* cells (wt*) and *GAL1*-regulated overexpressing *UBP10 ubp1Δ* (*1Δ*) to *ubp17Δ* (*17Δ*) single mutant cells either reppressed (OFF) or induced (ON) for Ubp10 overexpression, after a 90 minutes treatment with 0.020% MMS. TCA-obtained cells extracts were processed for immunoblotting with α-PCNA antibody.
(JPG)

Figure S4 Analysis of MMS sensitivity and PCNA ubiquitylation in *GAL1*-regulated overexpressing *UBP1, UBP2, UBP3*, *UBP4*, *UBP5, UBP6, UBP7, UBP8, UBP9, UBP10, UBP11, UBP12, UBP13, UBP14, UBP15, UBP16 and UBP17* yeast cells. (A) Ten-fold dilutions of equal numbers of wild-type and *GAL1,10*-expressing *UBP1, UBP2, UBP3*, *UBP4*, *UBP5, UBP6, UBP7, UBP8, UBP9, UBP10, UBP11, UBP12, UBP13, UBP14, UBP15, UBP16 and UBP17* cells were incubated at 25°C in the absence or in the chronic presence of MMS (as indicated) for 72 hours and photographed. (B) Immunodetection of modified PCNA forms in wild-type or *GAL1,10*-expressing *UBP1, UBP2, UBP3*, *UBP4*, *UBP5, UBP6, UBP7, UBP8, UBP9, UBP10, UBP11, UBP12, UBP13, UBP14, UBP15, UBP16 and UBP17* cells, after a 90 minutes treatment with 0.020% MMS. Cells extracts were processed for immunoblotting with α-PCNA antibodiy. Ponceau staining of the blotted protein extracts is shown for loading control.
(JPG)

Figure S5 Epistasis analysis of *pol30^K164R^* and *UBP10* mutant alleles. (A) Tenfold serial dilutions of wild-type, *pol30^K164R^*, *ubp10Δ* and *ubp10Δ pol30^K164R^* cells incubated at 25°C on YPAD plates with or without the indicated percentages of MMS for 72 hours and photographed. (B) Tenfold dilutions of equal numbers of (otherwise isogenic) wild-type, *pol30^K164R^*, *GAL1,10:UBP10* and *GAL1,10:UBP10 pol30^K164R^* cells incubated at 25°C on YAPD plates (GAL OFF) to repress *GAL1,10*-driven *UBP10* expression or YAPGal plates (GAL ON) to induce *GAL1*-driven *UBP10* expression (with or without MMS, as indicated).
(JPG)

Figure S6 Forward mutation analysis in wild-type and *GAL1,10:UBP10* strains. Canavanine resistance was assayed in wild-type, *GAL1,10:UBP10*, rev3*Δ*, and *GAL1,10:UBP10 rev3Δ* cells either incubated in galactose to induced *UBP10* overexpression (GAL ON) or in glucose to repress it (*UBP10* expression) and treated with 0.0005% MMS. Note that a low concentration of MMS was used in this assay because of the hypersensitivity of *UBP10* overexpressing cells (as shown in Figure S4B) to the DNA alkylating chemical. For the same reason, in these experiments a 56 hours exposure to the chemical was required for cultures to reach saturation (before plating onto canavanine Petri dishes). Plots of the resulting forward mutation frequencies are shown.
(JPG)

Figure S7 Catalytically active Ubp10 deubiquitylates PCNA *in vivo* independently from histone H2B deubiquitylation. (A) Ten-fold dilutions of equal numbers of wild-type, *ubp10Δ*, *GAL1,10:GST-UBP10*, *GAL1,10:GST-ubp10^C371S^*, *GAL1,10:GST-UBP10 bre1Δ* and *GAL1,10:GST-ubp10^C371S^ bre1Δ* cells were incubated at 25°C in the absence or the presence of indicated percentages of MMS for 72 hours and photographed. (B) Catalytically active Ubp10 reverts PCNA ubiquitylation *in vivo* independently from *BRE1*. Immunodetection of ubiquitylated PCNA forms in wild-type cells and in cells reppressed (GAL OFF) or induced (GAL ON) for GST-Ubp10 or GST-Ubp10^CS^ expression, after a 90 minutes treatment with 0.020% MMS. Protein extracts were processed for immunoblotting with policlonal α-PCNA antibody. Ponceau staining of the blotted protein extracts is shown for loading control.
(JPG)

Figure S8 Ubp10 is required for rapid deubiquitylation after MMS-induced DNA damage. Asynchronously growing cultures of wild-type and (otherwise isogenic) *ubp10Δ* strains were incubated 60 minutes in the presence of 0.02% MMS, washed twice in fresh (pre-warmed) media and release in YAPD (in the absence of the alkylating chemical). Samples were taken at indicated intervals and processed for immunodetection of PCNA forms and Rad53 phosphorylation with α-PCNA and α-Rad53 antibodies. ubPCNA was quantitated, normalized and plotted.
(JPG)

Figure S9 Analysis of *ubp10-myc* and *ubp10^C371S^-myc* strains. (A) Asynchronously growing Ubp10-myc cells were blocked in G1 with α-factor and then released in fresh medium to analyze the quantity of Ubp10 through the cell cycle; additionally, Ubp10-myc asynchronous cells were treated with 0.020% MMS 90 minutes, 0.2 M HU 90 minutes or 150 Jm^−2^ UV light. TCA-extracted protein samples were collected for detection of Ubp10-myc, PCNA and Rad53. (B) The lack of deubiquiting activity of Ubp10^C371S^ does not alter the level of the protein, but it causes an accumulation of ubiquitinated PCNA forms in a similar way than the deletion of the *UBP10* gene. Wild-type (wt), *ubp10Δ*, *ubp10^C371S^-myc* (two different clones) and *UBP10-myc* cells were treated with 0,02% MMS during 90 minutes and TCA-extracted protein samples were processed for Western analysis (to detect Ubp10-myc, PCNA and Rad53), all along with an untreated wt sample (as indicated). Note that, while *UBP10* mutants (*ubp10Δ* and the two *ubp10^C371S^-myc* clones) accumulate more mono- and di-UbPCNA, the *ubp10-myc* strain has wild-type levels. (C) Ubp10 interacts *in vivo* with PCNA throughout the cell cycle. Co-immunoprecipitation assay showing physical interaction between Ubp10-myc and PCNA. PCNA was immunoprecipitated from untreated asynchronous (As), α-factor synchronyzed (G1), 30 minutes released S-phase (S) or 75 minutes released G2 (G2) cells. Blots were incubated with α-myc (to detect Ubp10-myc) or α-PCNA. Appropriate input (WCE) and mock-Ip controls are shown. (D) Ubp10 interacts *in vivo* with PCNA in undamaged and MMS-damaged cells. PCNA was immunoprecipitated from untreated asynchronous (As) or 0.02% MMS-treated cells. Blots were incubated either with α-myc (to detect Ubp10-myc) or α-PCNA. Input (WCE) and mock-Ip controls are shown.
(JPG)

Figure S10 The E3-ubiquitin ligase Rad18 and Ubp10 ubiquitin-specific protease interact physically *in vivo*. Co-immunoprecipitation assay showing physical interaction between Ubp10-myc and Rad18-Ha. Ubp10-myc was immunoprecipitated either from untreated (Asyn) or 0.02% MMS-treated cells (MMS), blots were incubated with α-myc (to detect Ubp10) or α-Ha (to detect Rad18-Ha) as indicated. Appropriate single tagged, input (WCE) and mock-Ip controls are shown.
(JPG)

Figure S11 Analysis of Rev1-PCNA interaction in *pol30^K164R^* cells in wild-type and *ubp10*Δ strains. Co-immunoprecipitation assay showing physical interaction between Rev1-myc and PCNA in *pol30^K164R^* cells. PCNA was immunoprecipitated either from untreated or from 0.020% MMS-treated cells, blots were incubated with α-myc (to detect Rev1) or α-FLAG (to detect PCNA). As indicated the strains used in this assays were *REV1-myc pol30^K164R^-FLAG* and *REV1-myc pol30^K164R^-FLAG ubp10Δ*. Note that the relative amount of immunoprecipitated Rev1-myc was similar in *UBP10* or *ubp10Δ* cells indicating that Rev1 interacts with unmodified PCNA (*pol30^K164R^*) and that this interaction is not enhanced in *ubp10Δ* mutants.
(JPG)

Figure S12 Co-immunoprecipitation assay showing physical interaction between Rev1-myc and PCNA-FLAG in MMS-treated cells. Cell extracts were prepared as for ChIPs (in the presence of the crosslinking agent formaldehyde, see methods). PCNA-FLAG was immunoprecipitated from 0.02% MMS-treated cells (45′ or 90′ samples) or α-factor blocked cells (as indicated), blots were incubated with α-myc (to detect Rev1) or α-FLAG (to detect PCNA). As indicated, the strains used in this assays were *REV1-myc POL30-FLAG* and *REV1-myc POL30-FLAG ubp10Δ*. Note that this is a representative Western blot of the experiments plotted in Figure 4C.
(JPG)

Figure S13 Detection of ubiquitylated PCNA forms in asynchronous cultures of wild-type and *ubp10Δ* cells by immunoprecipitation. Immunoprecipitation of FLAG-tagged PCNA from asynchronous (Asyn) or 0.02% MMS-treated (MMS) cultures. Samples were taken from exponentially growing cultures or 90 minutes MMS-treated cultures of *POL30-FLAG* (wild-type) and *POL30-FLAG ubp10Δ* (*ubp10Δ*) strains and processed for immunoprecipitation with α-FLAG. Immunoblots were incubated with α-PCNA (to detect unmodified and modified PCNA). Note the detection of ubiquitylated PCNA in untreated wild-type and *ubp10Δ* cells, and the accumulation of ubiquitylated forms of PCNA in untreated and MMS-treated *ubp10*Δ cells (compared to wild-type samples).
(JPG)

Figure S14 Forward mutation analysis in wild-type and *ubp10Δ* strains. (A) Canavanine resistance was assayed in *ubp10Δ*, *ubp10Δ rev3Δ* and wild-type control cells either untreated or treated with 0.002% or 0.005% MMS (as indicated). Plots of the resulting forward mutation frequencies are shown. (B) Viability analysis in wild-type, *rev3Δ* and *ubp10Δ* strains. Exponentially growing wild-type, *rev3Δ* and *ubp10Δ* strains were exposed the indicated times to 0.05% or 0.2% MMS and test for colony formation. Plots of the resulting viability test are shown.
(JPG)

Table S1 Yeast strains used in this study.
(DOC)

## References

[1] Friedberg EC (2005). Suffering in silence: the tolerance of DNA damage.. Nat Rev Mol Cell Biol.

[2] Bergink S, Jentsch S (2009). Principles of ubiquitin and SUMO modifications in DNA repair.. Nature.

[3] Andersen PL, Xu F, Xiao W (2008). Eukaryotic DNA damage tolerance and translesion synthesis through covalent modifications of PCNA.. Cell Res.

[4] Chang DJ, Cimprich KA (2009). DNA damage tolerance: when it's OK to make mistakes.. Nat Chem Biol.

[5] Ulrich HD (2009). Regulating post-translational modifications of the eukaryotic replication clamp PCNA.. DNA Repair.

[6] Branzei D, Foiani M (2010). Maintaining genome stability at the replication fork.. Nat Rev Mol Cell Biol.

[7] Daigaku Y, Davies AA, Ulrich HD (2010). Ubiquitin-dependent DNA damage bypass is separable from genome replication.. Nature.

[8] Gallego-Sánchez A, Conde F, San-Segundo PA, Bueno A (2010). Control of PCNA deubiquitylation in yeast.. Biochem Soc Trans.

[9] Moldovan G-L, Pfander B, Jentsch S (2007). PCNA, the maestro of the replication fork.. Cell.

[10] Sabbioneda S, Gourdin AM, Green CM, Zotter A, Giglia-Mari G (2008). Effect of proliferating cell nuclear antigen ubiquitination and chromatin structure on the dynamic properties of the Y-family DNA polymerases.. Mol Biol Cell.

[11] Gan GN, Wittschieben JP, Wittschieben BØ, Wood RD (2008). DNA polymerase zeta (pol zeta) in higher eukaryotes.. Cell Res.

[12] Johnson RE, Washington MT, Haracska L, Prakash S, Prakash L (2000). Eukaryotic polymerases iota and zeta act sequentially to bypass DNA lesions.. Nature.

[13] Prakash S, Johnson RE, Prakash L (2005). Eukaryotic translesion synthesis DNA polymerases: specificity of structure and function.. Annu Rev Biochem.

[14] Nelson JR, Lawrence CW, Hinkle DC (1996). Thymine-thymine dimer bypass by yeast DNA polymerase zeta.. Science.

[15] Johnson RE, Washington MT, Prakash S, Prakash L (2000). Fidelity of human DNA polymerase eta.. J Biol Chem.

[16] Guo D, Wu X, Rajpal DK, Taylor JS, Wang Z (2001). Translesion synthesis by yeast DNA polymerase zeta from templates containing lesions of ultraviolet radiation and acetylaminofluorene.. Nucleic Acids Res.

[17] Haracska L, Prakash S, Prakash L (2003). Yeast DNA polymerase zeta is an efficient extender of primer ends opposite from 7,8-dihydro-8-Oxoguanine and O6-methylguanine.. Mol Cell Biol.

[18] Huang TT, Nijman SMB, Mirchandani KD, Galardy PJ, Cohn MA (2006). Regulation of monoubiquitinated PCNA by DUB autocleavage.. Nat Cell Biol.

[19] Huang TT, D'Andrea AD (2006). Regulation of DNA repair by ubiquitylation.. Nat Rev Mol Cell Biol.

[20] Brown S, Niimi A, Lehmann AR (2009). Ubiquitination and deubiquitination of PCNA in response to stalling of the replication fork.. Cell Cycle.

[21] Wilkinson KD (1997). Regulation of ubiquitin-dependent processes by deubiquitinating enzymes.. FASEB J.

[22] Bilsland E, Hult M, Bell SD, Sunnerhagen P, Downs JA (2007). The Bre5/Ubp3 ubiquitin protease complex from budding yeast contributes to the cellular response to DNA damage.. DNA Repair.

[23] Kvint K, Uhler JP, Taschner MJ, Sigurdsson S, Erdjument-Bromage H (2008). Reversal of RNA polymerase II ubiquitylation by the ubiquitin protease Ubp3.. Mol Cell.

[24] Emre NCT, Ingvarsdottir K, Wyce A, Wood A, Krogan NJ (2005). Maintenance of low histone ubiquitylation by Ubp10 correlates with telomere-proximal Sir2 association and gene silencing.. Mol Cell.

[25] Gardner RG, Nelson ZW, Gottschling DE (2005). Ubp10/Dot4p regulates the persistence of ubiquitinated histone H2B: distinct roles in telomeric silencing and general chromatin.. Mol Cell Biol.

[26] Schulze JM, Hentrich T, Nakanishi S, Gupta A, Emberly E (2011). Splitting the task: Ubp8 and Ubp10 deubiquitinate different cellular pools of H2BK123.. Genes Dev.

[27] Davies AA, Huttner D, Daigaku Y, Chen S, Ulrich HD (2008). Activation of ubiquitin-dependent DNA damage bypass is mediated by replication protein A.. Mol Cell.

[28] Karras GI, Jentsch S (2010). The RAD6 DNA damage tolerance pathway operates uncoupled from the replication fork and is functional beyond S phase.. Cell.

[29] Hoege C, Pfander B, Moldovan G-L, Pyrowolakis G, Jentsch S (2002). RAD6-dependent DNA repair is linked to modification of PCNA by ubiquitin and SUMO.. Nature.

[30] Hwang WW, Venkatasubrahmanyam S, Ianculescu AG, Tong A, Boone C (2003). A conserved RING finger protein required for histone H2B monoubiquitination and cell size control.. Mol Cell.

[31] Wood A, Krogan NJ, Dover J, Schneider J, Heidt J (2003). Bre1, an E3 ubiquitin ligase required for recruitment and substrate selection of Rad6 at a promoter.. Mol Cell.

[32] Paulovich AG, Hartwell LH (1995). A checkpoint regulates the rate of progression through S phase in S. cerevisiae in response to DNA damage.. Cell.

[33] Pellicioli A, Lucca C, Liberi G, Marini F, Lopes M (1999). Activation of Rad53 kinase in response to DNA damage and its effect in modulating phosphorylation of the lagging strand DNA polymerase.. EMBO J.

[34] Tercero JA, Diffley JF (2001). Regulation of DNA replication fork progression through damaged DNA by the Mec1/Rad53 checkpoint.. Nature.

[35] Tercero JA, Longhese MP, Diffley JFX (2003). A central role for DNA replication forks in checkpoint activation and response.. Mol Cell.

[36] Ideguchi H, Ueda A, Tanaka M, Yang J, Tsuji T (2002). Structural and functional characterization of the USP11 deubiquitinating enzyme, which interacts with the RanGTP-associated protein RanBPM.. Biochem J.

[37] Wu X, Yen L, Irwin L, Sweeney C, Carraway KL (2004). Stabilization of the E3 ubiquitin ligase Nrdp1 by the deubiquitinating enzyme USP8.. Mol Cell Biol.

[38] Kee Y, Lyon N, Huibregtse JM (2005). The Rsp5 ubiquitin ligase is coupled to and antagonized by the Ubp2 deubiquitinating enzyme.. EMBO J.

[39] Haracska L, Kondratick CM, Unk I, Prakash S, Prakash L (2001). Interaction with PCNA is essential for yeast DNA polymerase eta function.. Mol Cell.

[40] Garg P, Burgers PM (2005). Ubiquitinated proliferating cell nuclear antigen activates translesion DNA polymerases eta and REV1.. Proc Natl Acad Sci USA.

[41] Wood A, Garg P, Burgers PMJ (2007). A ubiquitin-binding motif in the translesion DNA polymerase Rev1 mediates its essential functional interaction with ubiquitinated proliferating cell nuclear antigen in response to DNA damage.. J Biol Chem.

[42] Chen C, Kolodner RD (1999). Gross chromosomal rearrangements in Saccharomyces cerevisiae replication and recombination defective mutants.. Nat Genet.

[43] Acharya N, Haracska L, Johnson RE, Unk I, Prakash S (2005). Complex formation of yeast Rev1 and Rev7 proteins: a novel role for the polymerase-associated domain.. Mol Cell Biol.

[44] Acharya N, Johnson RE, Prakash S, Prakash L (2006). Complex formation with Rev1 enhances the proficiency of Saccharomyces cerevisiae DNA polymerase zeta for mismatch extension and for extension opposite from DNA lesions.. Mol Cell Biol.

[45] Lawrence CW, Nisson PE, Christensen RB (1985). UV and chemical mutagenesis in rev7 mutants of yeast.. Mol Gen Genet.

[46] Zhao X, Muller EG, Rothstein R (1998). A suppressor of two essential checkpoint genes identifies a novel protein that negatively affects dNTP pools.. Mol Cell.

[47] Epstein CB, Cross FR (1992). CLB5: a novel B cyclin from budding yeast with a role in S phase.. Genes Dev.

[48] Kühne C, Linder P (1993). A new pair of B-type cyclins from Saccharomyces cerevisiae that function early in the cell cycle.. EMBO J.

[49] Schwob E, Nasmyth K (1993). CLB5 and CLB6, a new pair of B cyclins involved in DNA replication in Saccharomyces cerevisiae.. Genes Dev.

[50] Wilmes GM, Archambault V, Austin RJ, Jacobson MD, Bell SP (2004). Interaction of the S-phase cyclin Clb5 with an “RXL” docking sequence in the initiator protein Orc6 provides an origin-localized replication control switch.. Genes Dev.

[51] Kim J, Roeder RG (2009). Direct Bre1-Paf1 complex interactions and RING finger-independent Bre1-Rad6 interactions mediate histone H2B ubiquitylation in yeast.. J Biol Chem.

[52] Sarkies P, Reams C, Simpson LJ, Sale JE (2010). Epigenetic instability due to defective replication of structured DNA.. Mol Cell.

[53] Kahana A, Gottschling DE (1999). DOT4 links silencing and cell growth in Saccharomyces cerevisiae.. Mol Cell Biol.

[54] Wan Y, Chiang J-H, Lin C-H, Arens CE, Saleem RA (2010). Histone chaperone Chz1p regulates H2B ubiquitination and subtelomeric anti-silencing.. Nucleic Acids Res.

[55] Kannouche PL, Lehmann AR (2004). Ubiquitination of PCNA and the polymerase switch in human cells.. Cell Cycle.

[56] Nijman SMB, Luna-Vargas MPA, Velds A, Brummelkamp TR, Dirac AMG (2005). A genomic and functional inventory of deubiquitinating enzymes.. Cell.

[57] Kim JM, Parmar K, Huang M, Weinstock DM, Ruit CA (2009). Inactivation of murine Usp1 results in genomic instability and a Fanconi anemia phenotype.. Developmental Cell.

[58] Brun J, Chiu RK, Wouters BG, Gray DA (2010). Regulation of PCNA polyubiquitination in human cells.. BMC Res Notes.

[59] Kow YW, Bao G, Minesinger B, Jinks-Robertson S, Siede W (2005). Mutagenic effects of abasic and oxidized abasic lesions in Saccharomyces cerevisiae.. Nucleic Acids Res.

[60] Acharya N, Brahma A, Haracska L, Prakash L, Prakash S (2007). Mutations in the ubiquitin binding UBZ motif of DNA polymerase eta do not impair its function in translesion synthesis during replication.. Mol Cell Biol.

[61] Bao G, Kow YW (2009). Effect of sequence context and direction of replication on AP site bypass in Saccharomyces cerevisiae.. Mutat Res.

[62] Wiltrout ME, Walker GC (2011). Proteasomal regulation of the mutagenic translesion DNA polymerase, Saccharomyces cerevisiae Rev1.. DNA Repair.

[63] Waters LS, Walker GC (2006). The critical mutagenic translesion DNA polymerase Rev1 is highly expressed during G(2)/M phase rather than S phase.. Proc Natl Acad Sci USA.

[64] Sabbioneda S, Bortolomai I, Giannattasio M, Plevani P, Muzi-Falconi M (2007). Yeast Rev1 is cell cycle regulated, phosphorylated in response to DNA damage and its binding to chromosomes is dependent upon MEC1.. DNA Repair.

[65] Pagès V, Santa Maria SR, Prakash L, Prakash S (2009). Role of DNA damage-induced replication checkpoint in promoting lesion bypass by translesion synthesis in yeast.. Genes Dev.

[66] Göhler T, Sabbioneda S, Green CM, Lehmann AR (2011). ATR-mediated phosphorylation of DNA polymerase η is needed for efficient recovery from UV damage.. J Cell Biol.

[67] Pastushok L, Hanna M, Xiao W (2010). Constitutive fusion of ubiquitin to PCNA provides DNA damage tolerance independent of translesion polymerase activities.. Nucleic Acids Res.

[68] Sánchez M, Calzada A, Bueno A (1999). The Cdc6 protein is ubiquitinated in vivo for proteolysis in Saccharomyces cerevisiae.. J Biol Chem.

[69] Calzada A, Hodgson B, Kanemaki M, Bueno A, Labib K (2005). Molecular anatomy and regulation of a stable replisome at a paused eukaryotic DNA replication fork.. Genes Dev.

[70] Cordon-Preciado V, Ufano S, Bueno A (2006). Limiting amounts of budding yeast Rad53 S-phase checkpoint activity results in increased resistance to DNA alkylation damage.. Nucleic Acids Res.

[71] Conde F, Ontoso D, Acosta I, Gallego-Sánchez A, Bueno A (2010). Regulation of tolerance to DNA alkylating damage by Dot1 and Rad53 in Saccharomyces cerevisiae.. DNA Repair.

[72] Calzada A, Sacristán M, Sánchez E, Bueno A (2001). Cdc6 cooperates with Sic1 and Hct1 to inactivate mitotic cyclin-dependent kinases.. Nature.

[73] Hutter KJ, Eipel HE (1979). Microbial determinations by flow cytometry.. J Gen Microbiol.

[74] Conde F, San-Segundo PA (2008). Role of Dot1 in the response to alkylating DNA damage in Saccharomyces cerevisiae: regulation of DNA damage tolerance by the error-prone polymerases Polzeta/Rev1.. Genetics.

[75] Longtine MS, McKenzie A, Demarini DJ, Shah NG, Wach A (1998). Additional modules for versatile and economical PCR-based gene deletion and modification in Saccharomyces cerevisiae.. Yeast.

[76] Longhese MP, Paciotti V, Fraschini R, Zaccarini R, Plevani P (1997). The novel DNA damage checkpoint protein ddc1p is phosphorylated periodically during the cell cycle and in response to DNA damage in budding yeast.. EMBO J.

[77] Calzada A, Sánchez M, Sánchez E, Bueno A (2000). The stability of the Cdc6 protein is regulated by cyclin-dependent kinase/cyclin B complexes in Saccharomyces cerevisiae.. J Biol Chem.

[78] Kanemaki M, Labib K (2006). Distinct roles for Sld3 and GINS during establishment and progression of eukaryotic DNA replication forks.. EMBO J.

[79] San-Segundo PA, Roeder GS (1999). Pch2 links chromatin silencing to meiotic checkpoint control.. Cell.

