# Supplementary figures and images for: Reversal of PCNA Ubiquitylation by Ubp10 in Saccharomyces cerevisiae
Source: PLoS Genet. 2012 Jul 19;8(7):e1002826. doi: 10.1371/journal.pgen.1002826 (PMC3400564; doi:10.1371/journal.pgen.1002826)

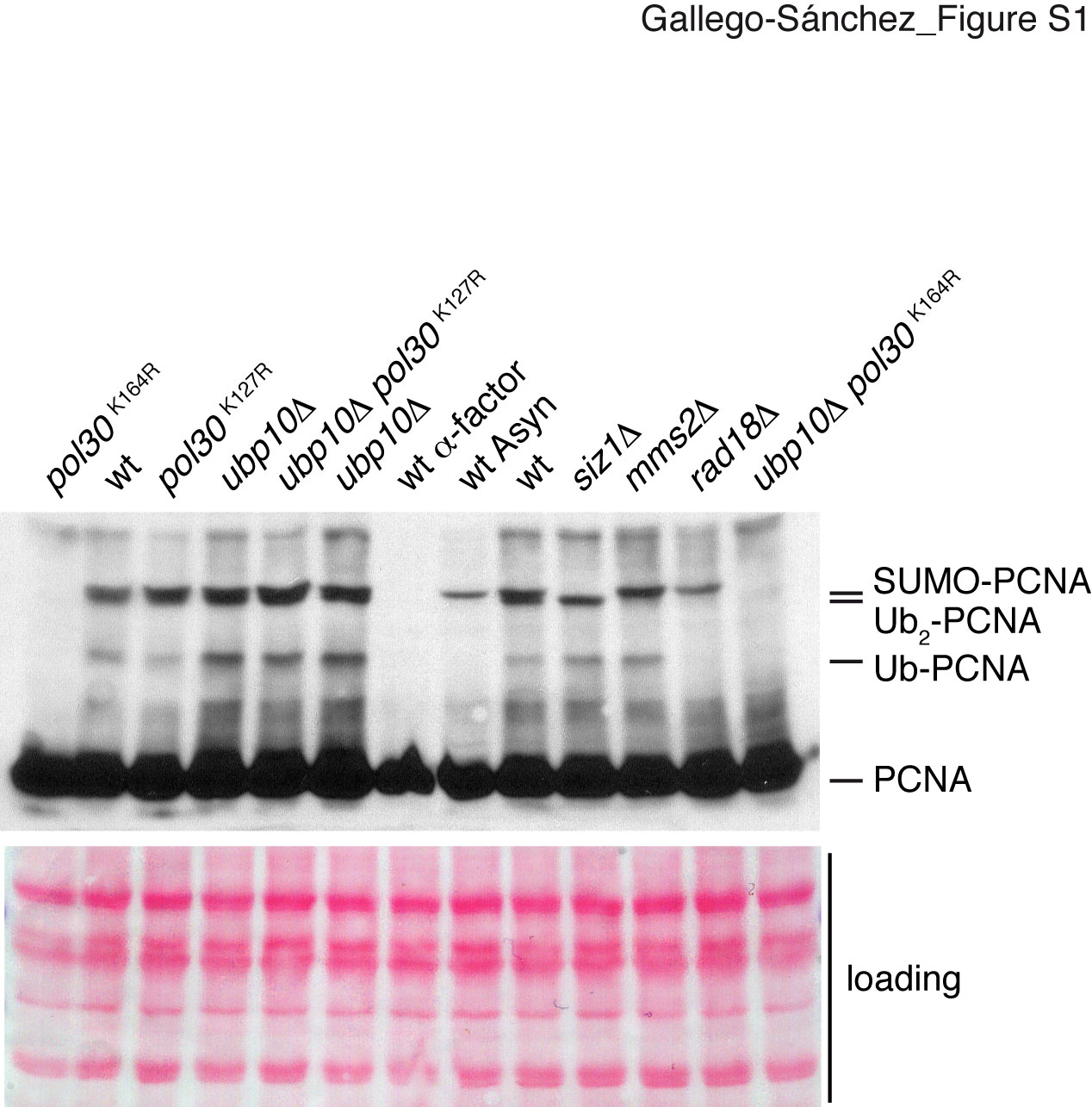

Supplement: Figure S1 — Immunodetection of ubiquitylated forms of PCNA in yeast TCA-cell extracts to show that UBP10 mutant cells accumulate K164 but not K127 modified PCNA forms. Immunoblot analysis with α-PCNA antibody of TCA-protein extracts from pol30K164R (unable to ubiquitylate or SUMOylate PCNA at K164), wild-type (wt), pol30K127R (unable to ubiquitylate or SUMOylate PCNA at K127), ubp10Δ, ubp10Δ pol30K127R, ubp10Δ, G1 wild-type (wt α-factor), siz1Δ (unable to SUMOylate PCNA), mms2Δ (unable to di-ubiquitylate PCNA), rad18Δ (unable to ubiquitylate PCNA), and ubp10Δ pol30K164R cells treated 90 minutes with 0.020% MMS and resolved in a 12% polyacrylamide gel, note the presence of a sample from untreated wild-type cells (8th lane). (JPG) [file pgen.1002826.s001.jpg]

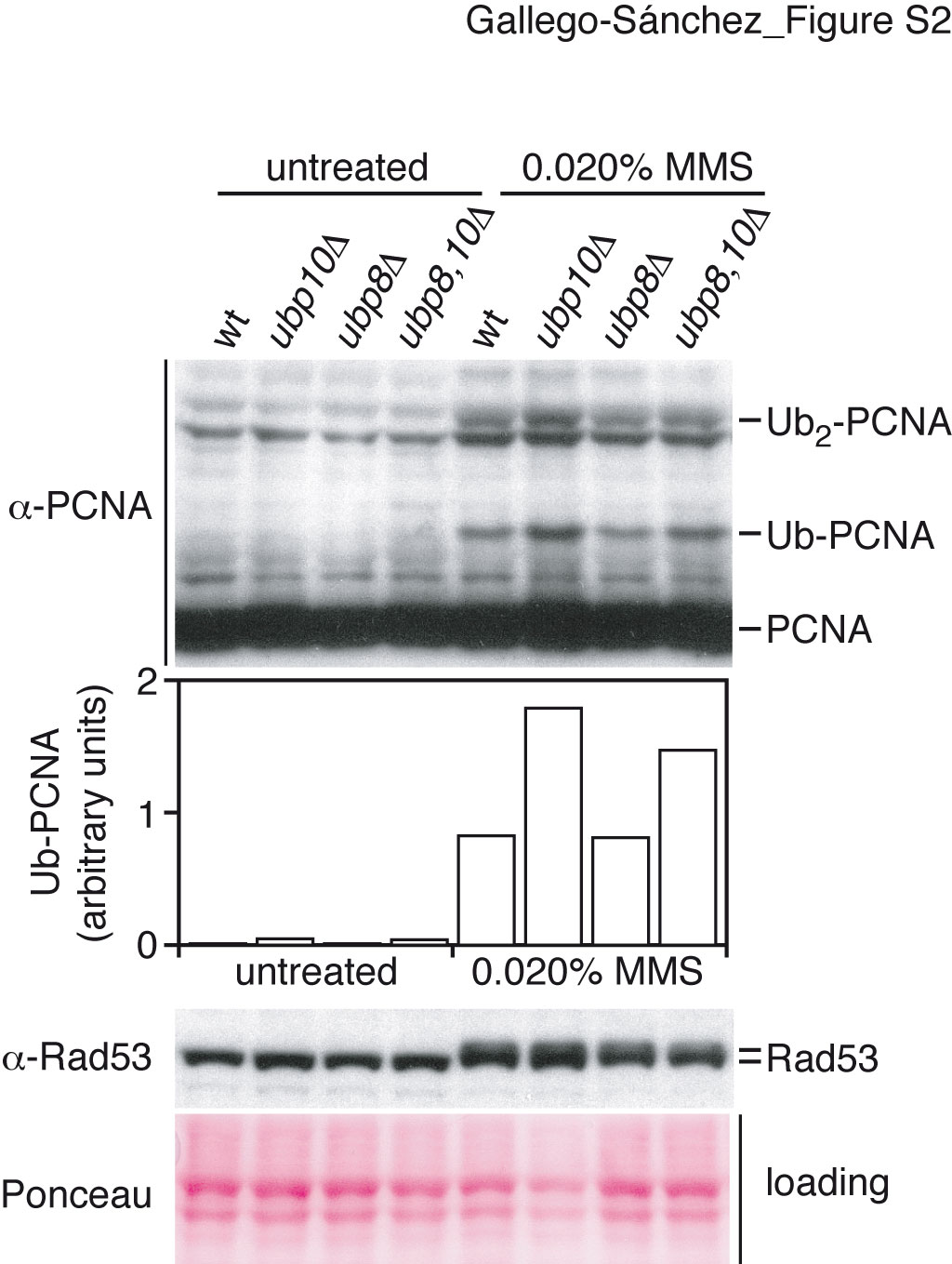

Supplement: Figure S2 — ubp10 but not ubp8 mutant cells accumulate ubiquitylated forms of PCNA in response to MMS-induced DNA damage. Immunodetection of mono-ubiquitylated (ubPCNA) and di-ubiquitylated PCNA (Ub2-PCNA) in wild-type, ubp8Δ, ubp10Δ and ubp8Δ ubp10Δ cells treated with 0.020% MMS (as indicated). Ubiquiylated PCNA (ubPCNA) samples were quantified, normalized to loading controls and plotted. Rad53 phosphorylation is used for testing checkpoint activation upon MMS-treatment. (JPG) [file pgen.1002826.s002.jpg]

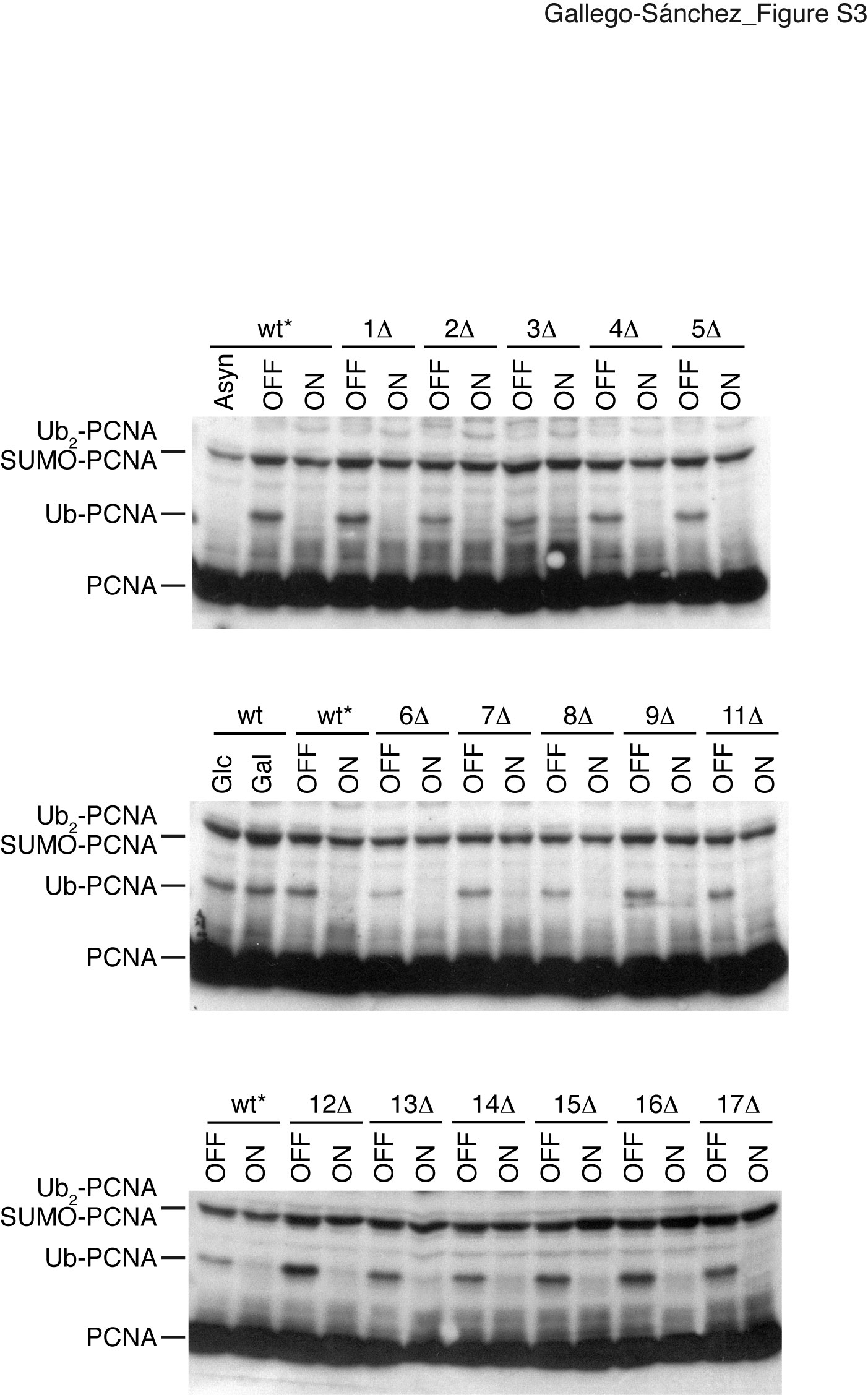

Supplement: Figure S3 — GAL1-driven overproduction of UBP10 reverts PCNA ubiquitylation in any UBP1-17 deletion. Catalytically active Ubp10 reverts PCNA ubiquitylation in vivo in ubp1Δ (1Δ) to ubp17Δ (17Δ) single mutants. Immunodetection of K164-monoubiquitylated PCNA forms in wild-type cells (wt), GAL1-regulated overexpressing UBP10 cells (wt*) and GAL1-regulated overexpressing UBP10 ubp1Δ (1Δ) to ubp17Δ (17Δ) single mutant cells either reppressed (OFF) or induced (ON) for Ubp10 overexpression, after a 90 minutes treatment with 0.020% MMS. TCA-obtained cells extracts were processed for immunoblotting with α-PCNA antibody. (JPG) [file pgen.1002826.s003.jpg]

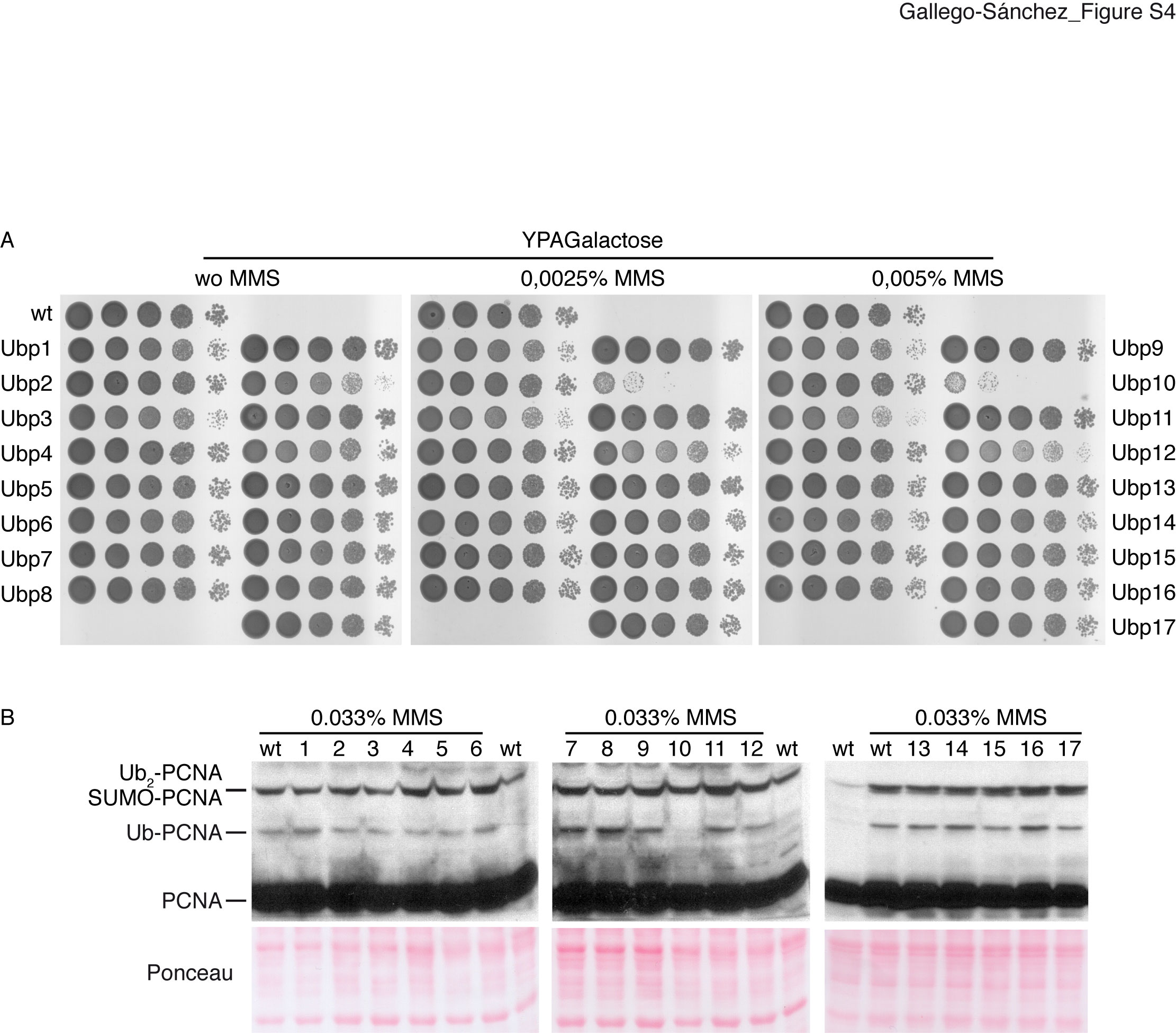

Supplement: Figure S4 — Analysis of MMS sensitivity and PCNA ubiquitylation in GAL1-regulated overexpressing UBP1, UBP2, UBP3, UBP4, UBP5, UBP6, UBP7, UBP8, UBP9, UBP10, UBP11, UBP12, UBP13, UBP14, UBP15, UBP16 and UBP17 yeast cells. (A) Ten-fold dilutions of equal numbers of wild-type and GAL1,10-expressing UBP1, UBP2, UBP3, UBP4, UBP5, UBP6, UBP7, UBP8, UBP9, UBP10, UBP11, UBP12, UBP13, UBP14, UBP15, UBP16 and UBP17 cells were incubated at 25°C in the absence or in the chronic presence of MMS (as indicated) for 72 hours and photographed. (B) Immunodetection of modified PCNA forms in wild-type or GAL1,10-expressing UBP1, UBP2, UBP3, UBP4, UBP5, UBP6, UBP7, UBP8, UBP9, UBP10, UBP11, UBP12, UBP13, UBP14, UBP15, UBP16 and UBP17 cells, after a 90 minutes treatment with 0.020% MMS. Cells extracts were processed for immunoblotting with α-PCNA antibodiy. Ponceau staining of the blotted protein extracts is shown for loading control. (JPG) [file pgen.1002826.s004.jpg]

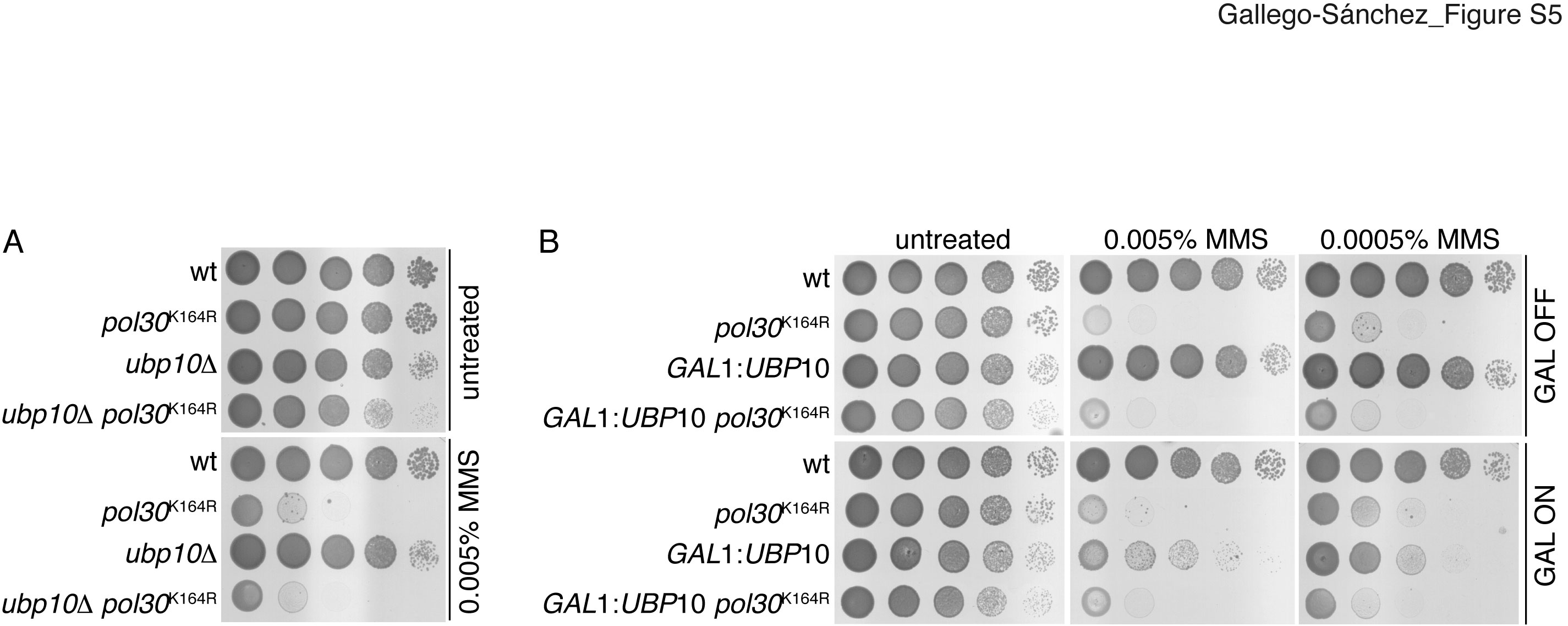

Supplement: Figure S5 — Epistasis analysis of pol30K164R and UBP10 mutant alleles. (A) Tenfold serial dilutions of wild-type, pol30K164R, ubp10Δ and ubp10Δ pol30K164R cells incubated at 25°C on YPAD plates with or without the indicated percentages of MMS for 72 hours and photographed. (B) Tenfold dilutions of equal numbers of (otherwise isogenic) wild-type, pol30K164R, GAL1,10:UBP10 and GAL1,10:UBP10 pol30K164R cells incubated at 25°C on YAPD plates (GAL OFF) to repress GAL1,10-driven UBP10 expression or YAPGal plates (GAL ON) to induce GAL1-driven UBP10 expression (with or without MMS, as indicated). (JPG) [file pgen.1002826.s005.jpg]

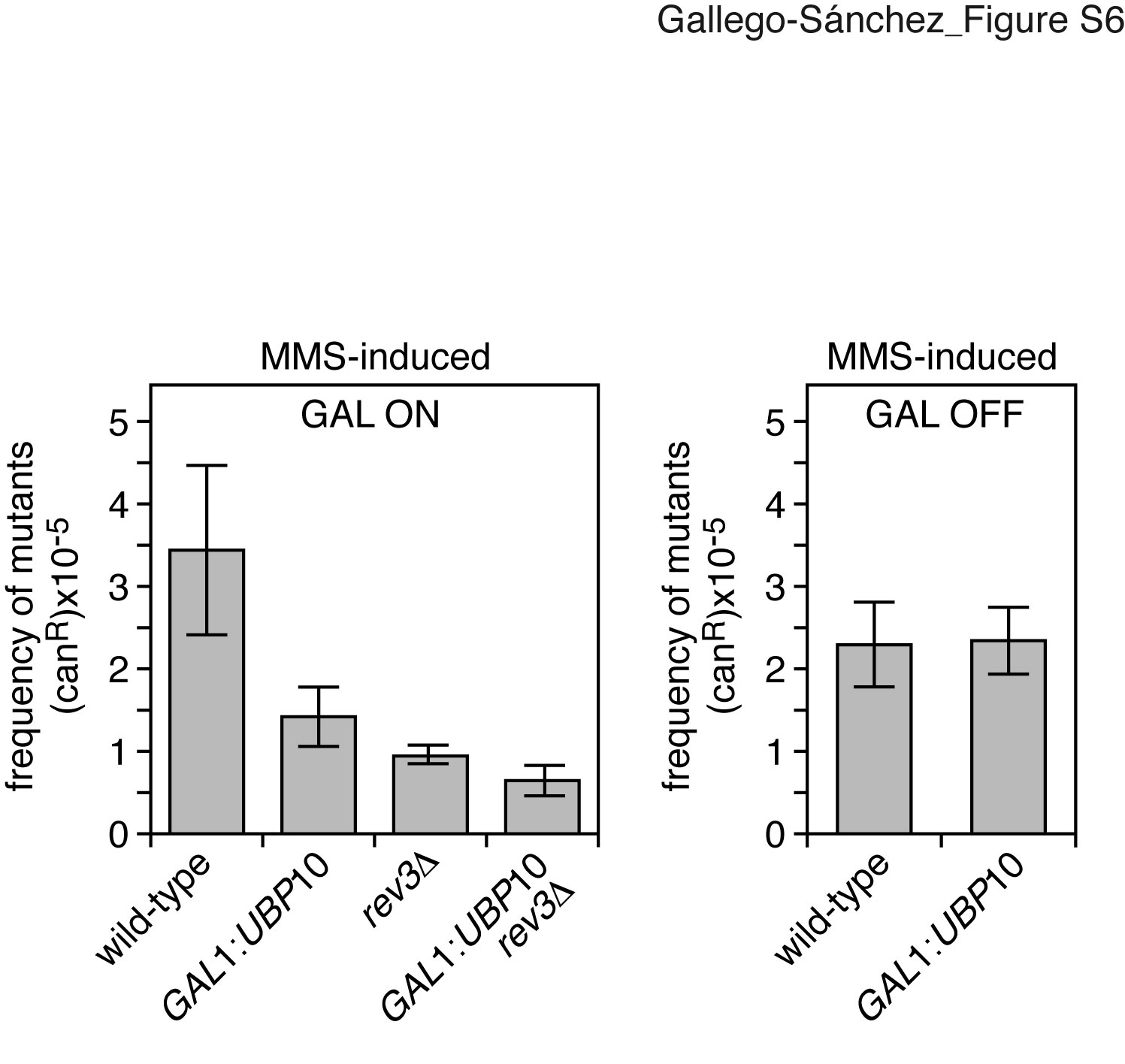

Supplement: Figure S6 — Forward mutation analysis in wild-type and GAL1,10:UBP10 strains. Canavanine resistance was assayed in wild-type, GAL1,10:UBP10, rev3Δ, and GAL1,10:UBP10 rev3Δ cells either incubated in galactose to induced UBP10 overexpression (GAL ON) or in glucose to repress it (UBP10 expression) and treated with 0.0005% MMS. Note that a low concentration of MMS was used in this assay because of the hypersensitivity of UBP10 overexpressing cells (as shown in Figure S4B) to the DNA alkylating chemical. For the same reason, in these experiments a 56 hours exposure to the chemical was required for cultures to reach saturation (before plating onto canavanine Petri dishes). Plots of the resulting forward mutation frequencies are shown. (JPG) [file pgen.1002826.s006.jpg]

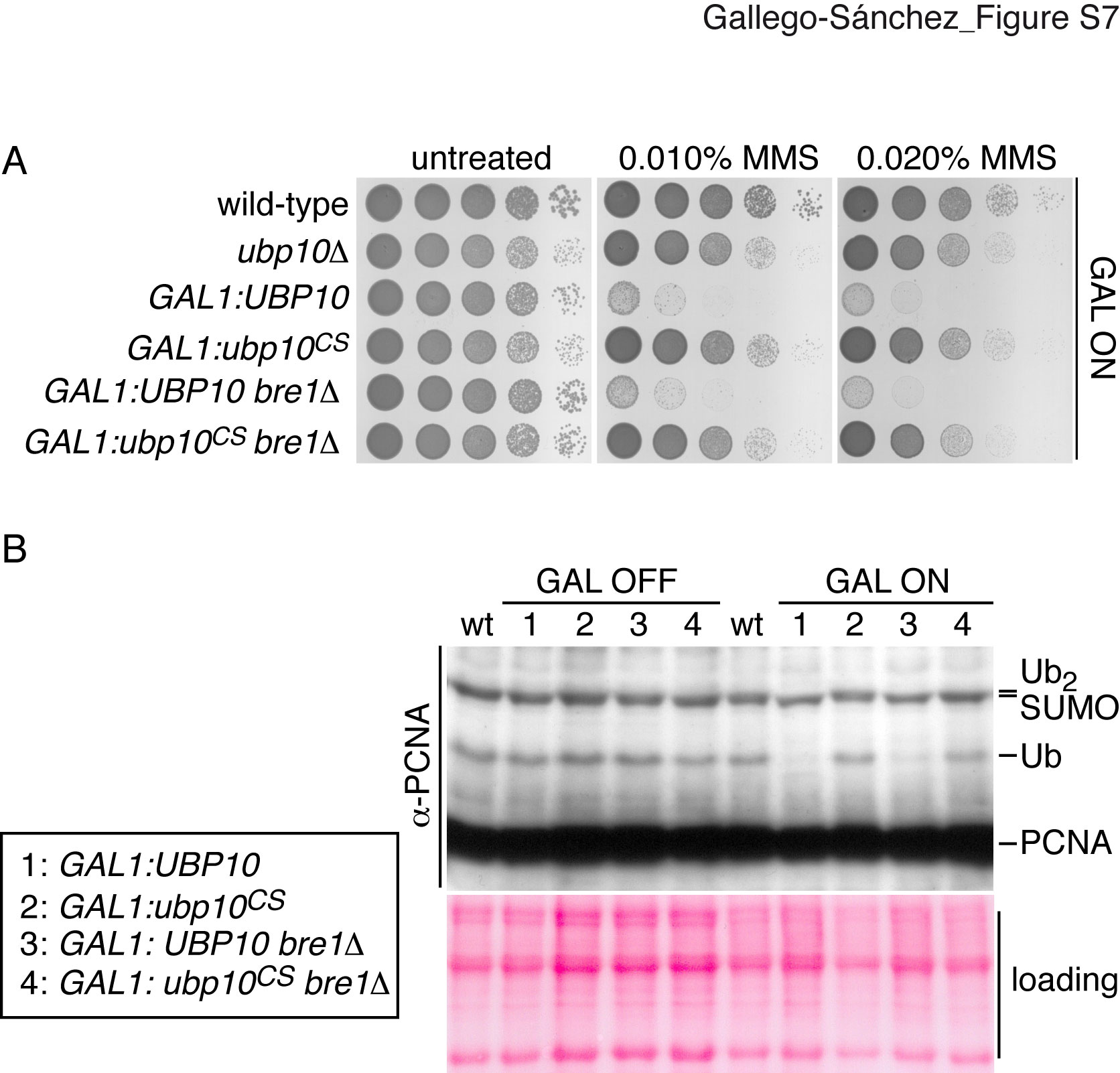

Supplement: Figure S7 — Catalytically active Ubp10 deubiquitylates PCNA in vivo independently from histone H2B deubiquitylation. (A) Ten-fold dilutions of equal numbers of wild-type, ubp10Δ, GAL1,10:GST-UBP10, GAL1,10:GST-ubp10C371S, GAL1,10:GST-UBP10 bre1Δ and GAL1,10:GST-ubp10C371S bre1Δ cells were incubated at 25°C in the absence or the presence of indicated percentages of MMS for 72 hours and photographed. (B) Catalytically active Ubp10 reverts PCNA ubiquitylation in vivo independently from BRE1. Immunodetection of ubiquitylated PCNA forms in wild-type cells and in cells reppressed (GAL OFF) or induced (GAL ON) for GST-Ubp10 or GST-Ubp10CS expression, after a 90 minutes treatment with 0.020% MMS. Protein extracts were processed for immunoblotting with policlonal α-PCNA antibody. Ponceau staining of the blotted protein extracts is shown for loading control. (JPG) [file pgen.1002826.s007.jpg]

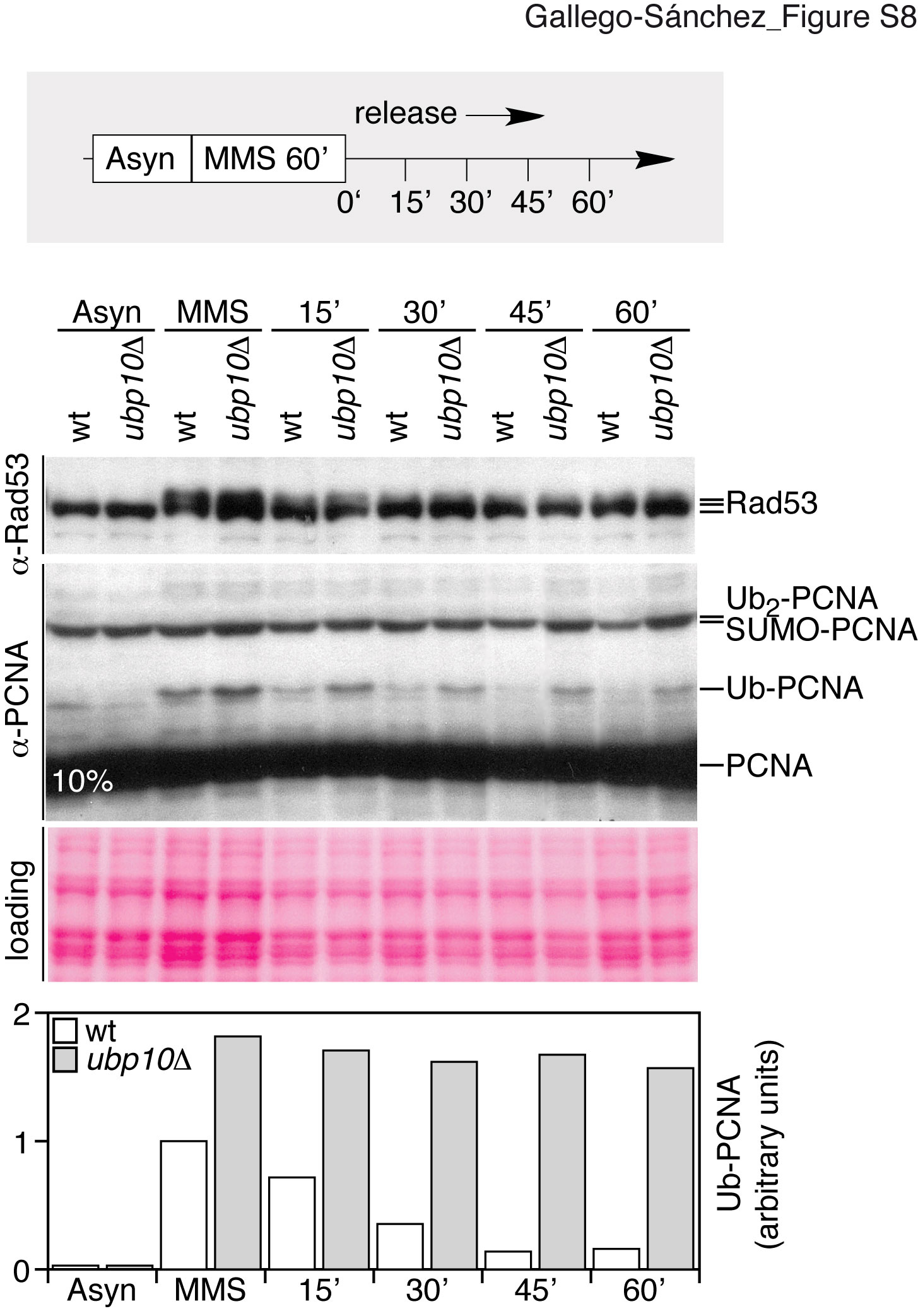

Supplement: Figure S8 — Ubp10 is required for rapid deubiquitylation after MMS-induced DNA damage. Asynchronously growing cultures of wild-type and (otherwise isogenic) ubp10Δ strains were incubated 60 minutes in the presence of 0.02% MMS, washed twice in fresh (pre-warmed) media and release in YAPD (in the absence of the alkylating chemical). Samples were taken at indicated intervals and processed for immunodetection of PCNA forms and Rad53 phosphorylation with α-PCNA and α-Rad53 antibodies. ubPCNA was quantitated, normalized and plotted. (JPG) [file pgen.1002826.s008.jpg]

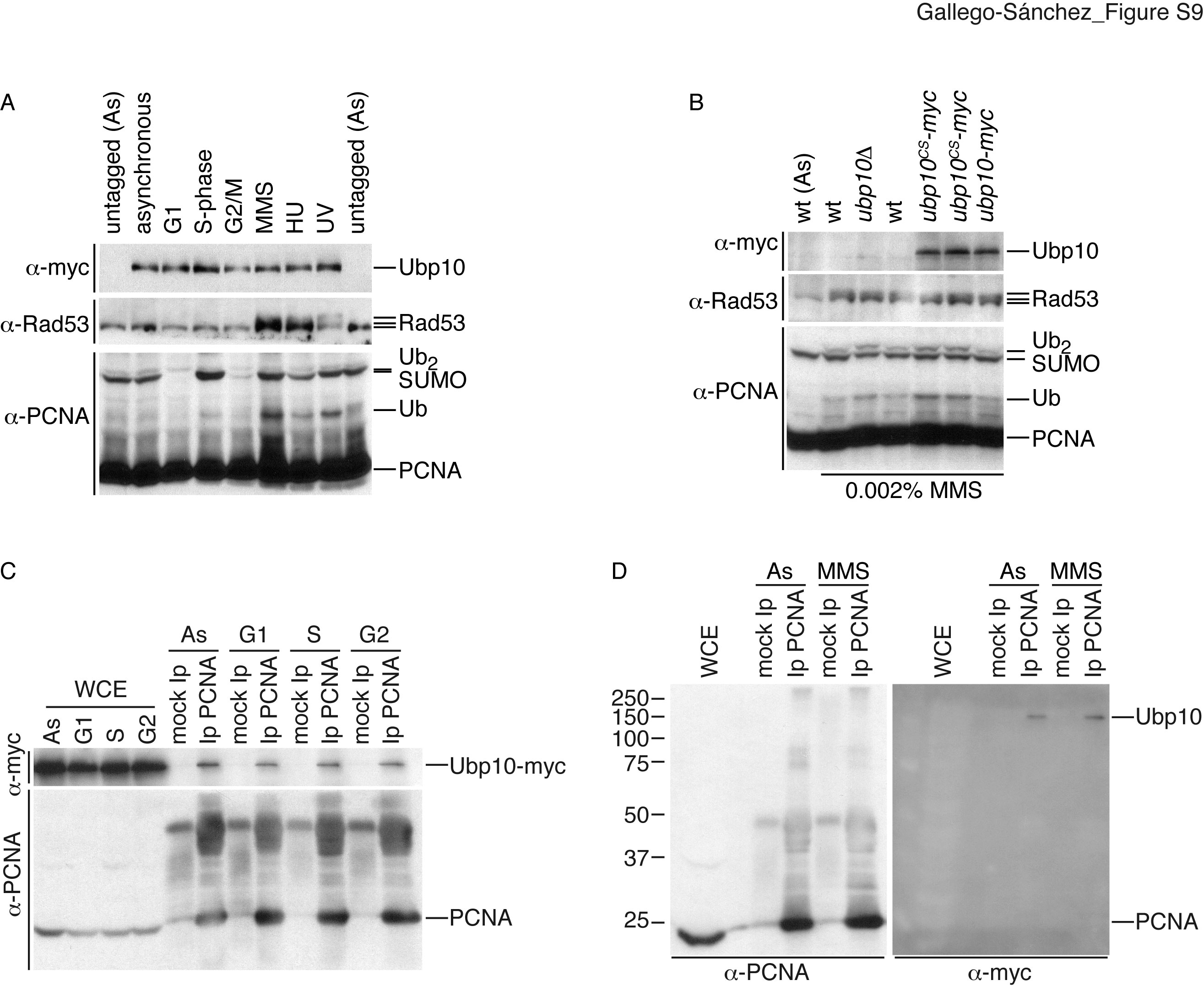

Supplement: Figure S9 — Analysis of ubp10-myc and ubp10C371S-myc strains. (A) Asynchronously growing Ubp10-myc cells were blocked in G1 with α-factor and then released in fresh medium to analyze the quantity of Ubp10 through the cell cycle; additionally, Ubp10-myc asynchronous cells were treated with 0.020% MMS 90 minutes, 0.2 M HU 90 minutes or 150 Jm−2 UV light. TCA-extracted protein samples were collected for detection of Ubp10-myc, PCNA and Rad53. (B) The lack of deubiquiting activity of Ubp10C371S does not alter the level of the protein, but it causes an accumulation of ubiquitinated PCNA forms in a similar way than the deletion of the UBP10 gene. Wild-type (wt), ubp10Δ, ubp10C371S-myc (two different clones) and UBP10-myc cells were treated with 0,02% MMS during 90 minutes and TCA-extracted protein samples were processed for Western analysis (to detect Ubp10-myc, PCNA and Rad53), all along with an untreated wt sample (as indicated). Note that, while UBP10 mutants (ubp10Δ and the two ubp10C371S-myc clones) accumulate more mono- and di-UbPCNA, the ubp10-myc strain has wild-type levels. (C) Ubp10 interacts in vivo with PCNA throughout the cell cycle. Co-immunoprecipitation assay showing physical interaction between Ubp10-myc and PCNA. PCNA was immunoprecipitated from untreated asynchronous (As), α-factor synchronyzed (G1), 30 minutes released S-phase (S) or 75 minutes released G2 (G2) cells. Blots were incubated with α-myc (to detect Ubp10-myc) or α-PCNA. Appropriate input (WCE) and mock-Ip controls are shown. (D) Ubp10 interacts in vivo with PCNA in undamaged and MMS-damaged cells. PCNA was immunoprecipitated from untreated asynchronous (As) or 0.02% MMS-treated cells. Blots were incubated either with α-myc (to detect Ubp10-myc) or α-PCNA. Input (WCE) and mock-Ip controls are shown. (JPG) [file pgen.1002826.s009.jpg]

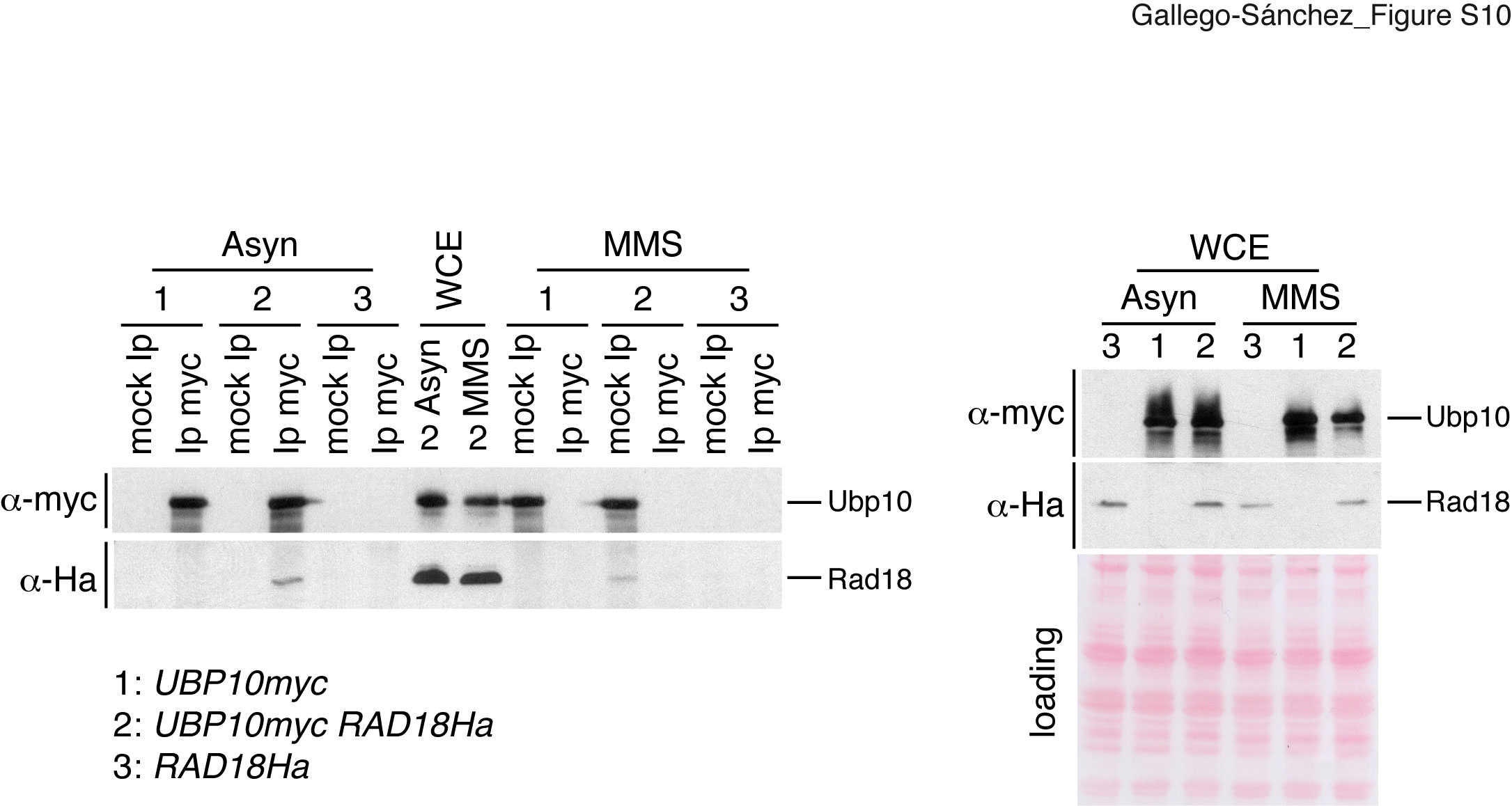

Supplement: Figure S10 — The E3-ubiquitin ligase Rad18 and Ubp10 ubiquitin-specific protease interact physically in vivo. Co-immunoprecipitation assay showing physical interaction between Ubp10-myc and Rad18-Ha. Ubp10-myc was immunoprecipitated either from untreated (Asyn) or 0.02% MMS-treated cells (MMS), blots were incubated with α-myc (to detect Ubp10) or α-Ha (to detect Rad18-Ha) as indicated. Appropriate single tagged, input (WCE) and mock-Ip controls are shown. (JPG) [file pgen.1002826.s010.jpg]

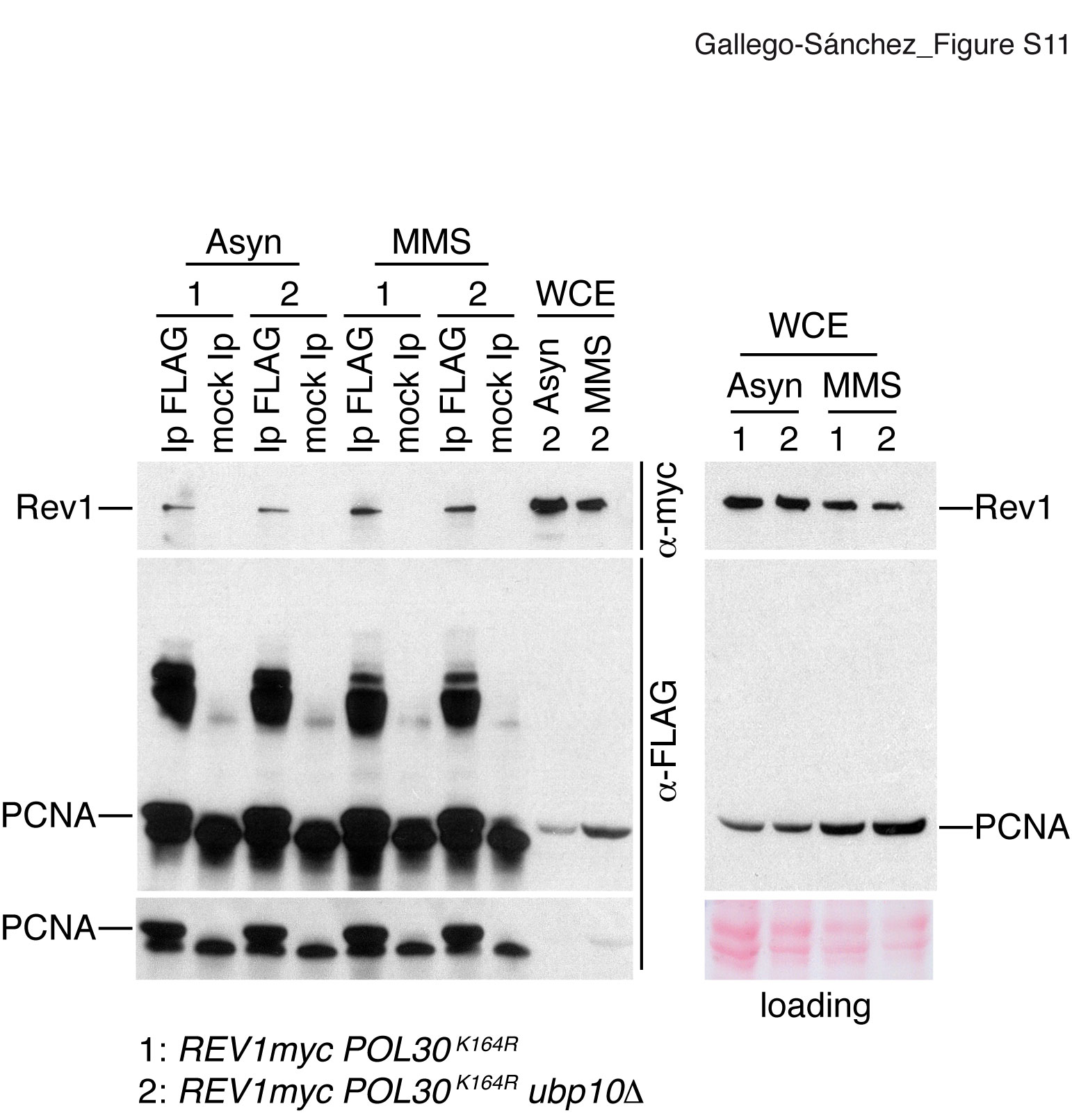

Supplement: Figure S11 — Analysis of Rev1-PCNA interaction in pol30K164R cells in wild-type and ubp10Δ strains. Co-immunoprecipitation assay showing physical interaction between Rev1-myc and PCNA in pol30K164R cells. PCNA was immunoprecipitated either from untreated or from 0.020% MMS-treated cells, blots were incubated with α-myc (to detect Rev1) or α-FLAG (to detect PCNA). As indicated the strains used in this assays were REV1-myc pol30K164R-FLAG and REV1-myc pol30K164R-FLAG ubp10Δ. Note that the relative amount of immunoprecipitated Rev1-myc was similar in UBP10 or ubp10Δ cells indicating that Rev1 interacts with unmodified PCNA (pol30K164R) and that this interaction is not enhanced in ubp10Δ mutants. (JPG) [file pgen.1002826.s011.jpg]

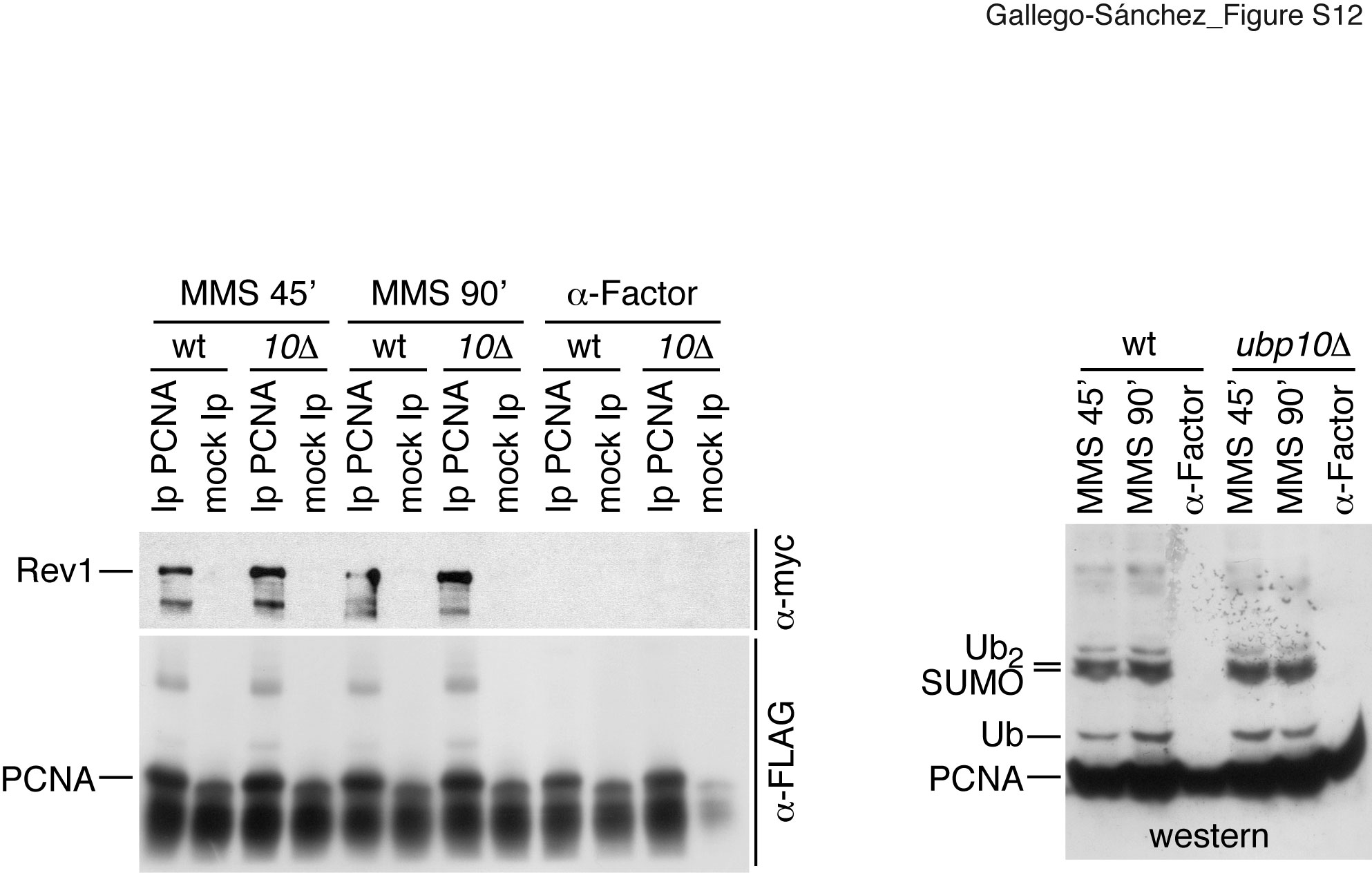

Supplement: Figure S12 — Co-immunoprecipitation assay showing physical interaction between Rev1-myc and PCNA-FLAG in MMS-treated cells. Cell extracts were prepared as for ChIPs (in the presence of the crosslinking agent formaldehyde, see methods). PCNA-FLAG was immunoprecipitated from 0.02% MMS-treated cells (45′ or 90′ samples) or α-factor blocked cells (as indicated), blots were incubated with α-myc (to detect Rev1) or α-FLAG (to detect PCNA). As indicated, the strains used in this assays were REV1-myc POL30-FLAG and REV1-myc POL30-FLAG ubp10Δ. Note that this is a representative Western blot of the experiments plotted in Figure 4C. (JPG) [file pgen.1002826.s012.jpg]

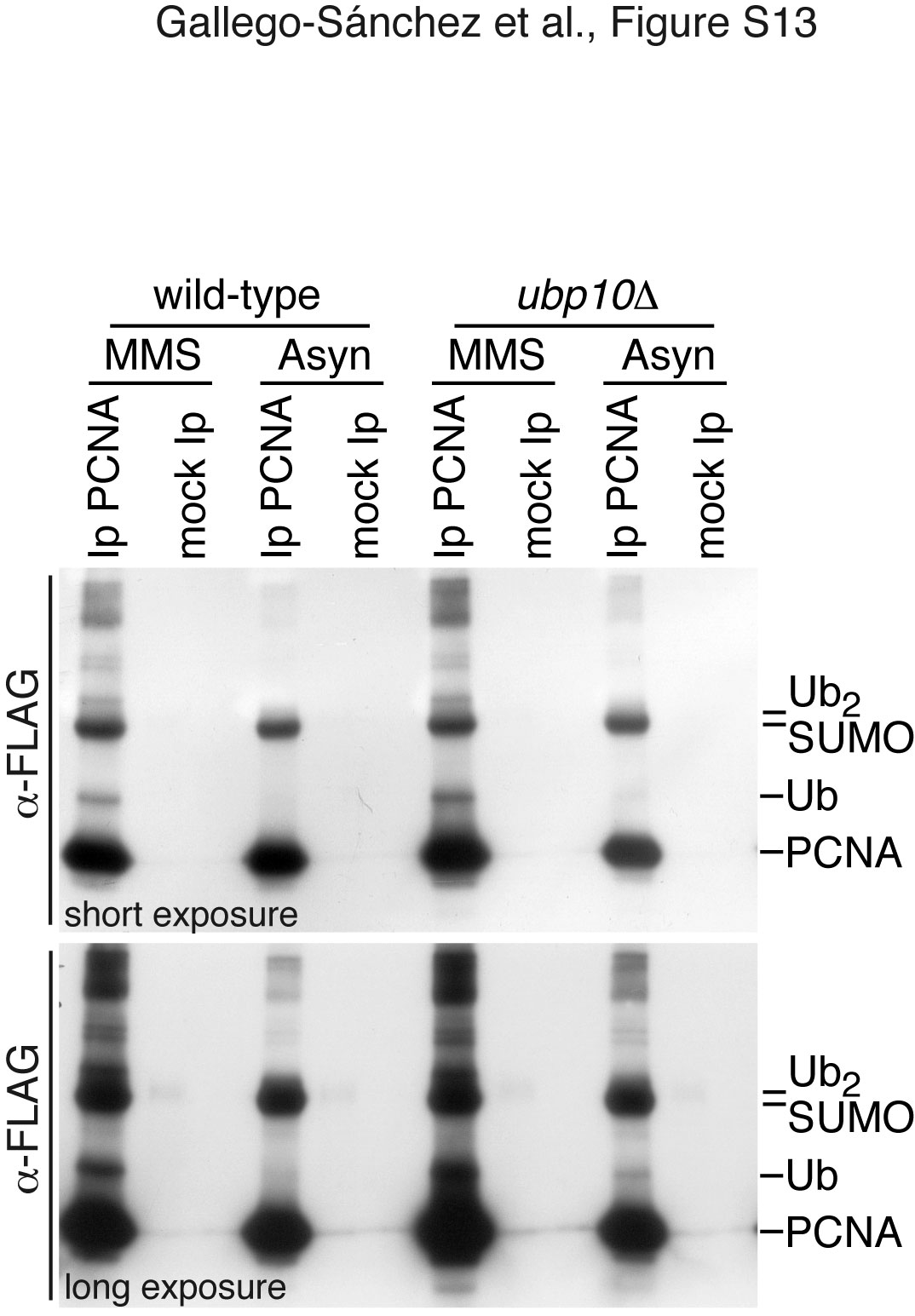

Supplement: Figure S13 — Detection of ubiquitylated PCNA forms in asynchronous cultures of wild-type and ubp10Δ cells by immunoprecipitation. Immunoprecipitation of FLAG-tagged PCNA from asynchronous (Asyn) or 0.02% MMS-treated (MMS) cultures. Samples were taken from exponentially growing cultures or 90 minutes MMS-treated cultures of POL30-FLAG (wild-type) and POL30-FLAG ubp10Δ (ubp10Δ) strains and processed for immunoprecipitation with α-FLAG. Immunoblots were incubated with α-PCNA (to detect unmodified and modified PCNA). Note the detection of ubiquitylated PCNA in untreated wild-type and ubp10Δ cells, and the accumulation of ubiquitylated forms of PCNA in untreated and MMS-treated ubp10Δ cells (compared to wild-type samples). (JPG) [file pgen.1002826.s013.jpg]

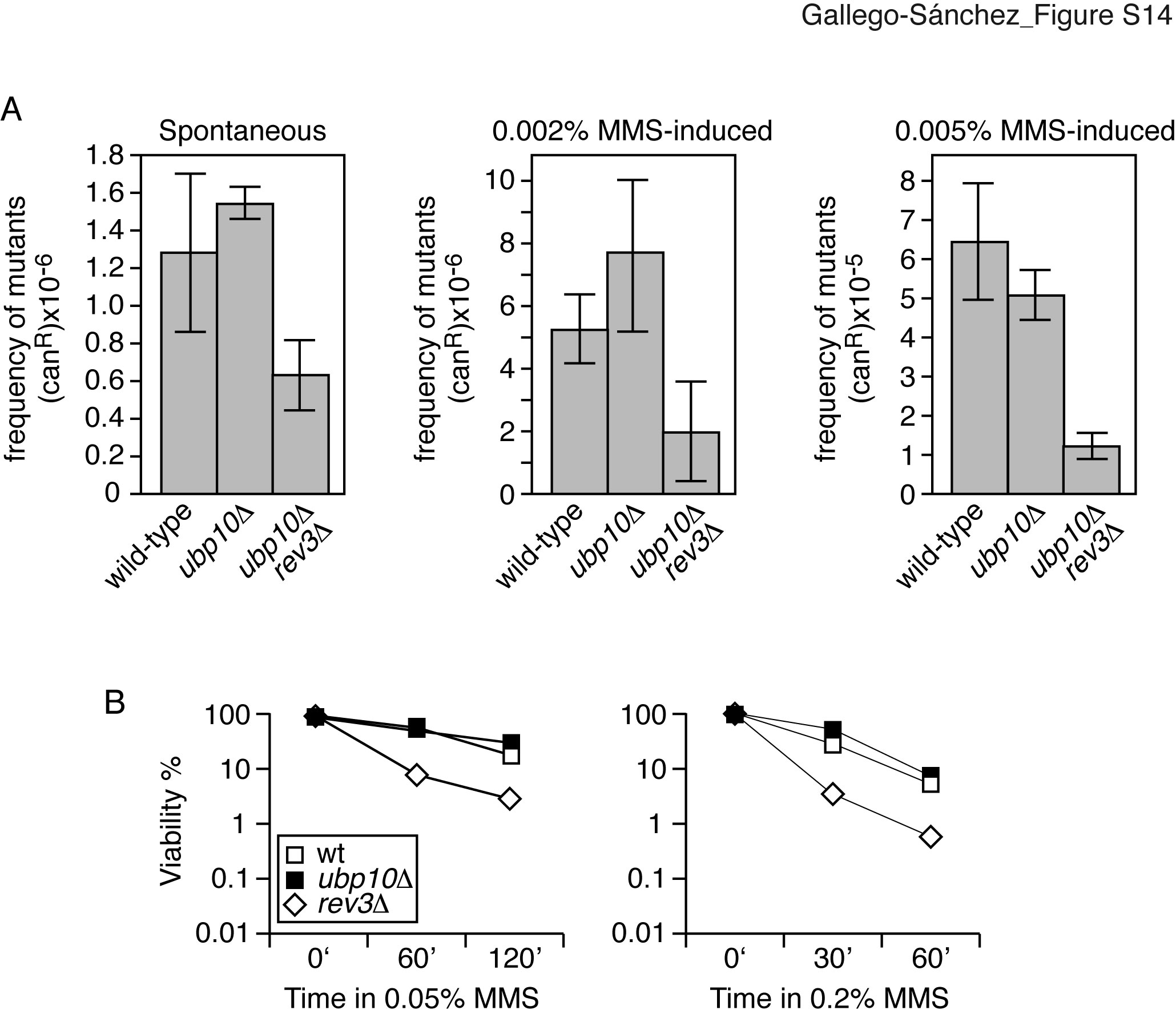

Supplement: Figure S14 — Forward mutation analysis in wild-type and ubp10Δ strains. (A) Canavanine resistance was assayed in ubp10Δ, ubp10Δ rev3Δ and wild-type control cells either untreated or treated with 0.002% or 0.005% MMS (as indicated). Plots of the resulting forward mutation frequencies are shown. (B) Viability analysis in wild-type, rev3Δ and ubp10Δ strains. Exponentially growing wild-type, rev3Δ and ubp10Δ strains were exposed the indicated times to 0.05% or 0.2% MMS and test for colony formation. Plots of the resulting viability test are shown. (JPG) [file pgen.1002826.s014.jpg]
