# Supplementary material for: Reversal of PCNA Ubiquitylation by Ubp10 in Saccharomyces cerevisiae
Source: PLoS Genet. 2012 Jul 19;8(7):e1002826. doi: 10.1371/journal.pgen.1002826 (PMC3400564; doi:10.1371/journal.pgen.1002826)
Supplement: Table S1 — Yeast strains used in this study. (DOC) [file pgen.1002826.s015.doc]

**Table S1** Gallego-Sánchez et al.,

Yeast strains used in this study. All indicated strains are isogenic with the W303 background.

| **Strain** | **Genotype** | **Origin** |
| --- | --- | --- |
| 55Sc34.08 | **W303** *Mata ade2-1 can1-100 his3-11,15 leu2-3,112 trp1-1 ura3-1 RAD5 bar1Δ::LEU2* | G.C. Walker |
| 61Sc33.09 | 55Sc34.08 with *ubp10Δ::HphMX4* | This work |
| 58Sc23.08 | 55Sc34.08 with *rad18Δ::KanMX6* | This work |
| 57Sc74.08 | 55Sc34.08 with *mms2Δ::HIS3* | This work |
| 58Sc25.08 | 55Sc34.08 with *siz1Δ::KanMX6* | This work |
| 55Sc36.08 | 55Sc34.08 with *rev3Δ::KanMX6* | G.C. Walker |
| 55Sc63.08 | 55Sc34.08 with *pol30^K164R^* | This work |
| 61Sc35.09 | 55Sc34.08 with *pol30^K164R^ ubp10::KanMX6* | This work |
| 60Sc62.09 | 55Sc34.08 with *KanMX6:GAL1,10:GST:ubp10* | This work |
| 60Sc61.09 | 55Sc34.08 with *KanMX6:GAL1,10:GST:ubp10^C371S^* | This work |
| 59Sc57.09 | 55Sc34.08 with *ubp10-13Myc:HphMX4* | This work |
| 61Sc45.10 | 55Sc34.08 with *pol30-3FLAG:KanMX6* | This work |
| 62Sc43.10 | 55Sc34.08 with *ubp10-13Myc:HphMX4 pol30-3FLAG:KanMX6* | This work |
| 62Sc34.10 | 55Sc34.08 with *ubp10-13Myc:HphMX4 rad18Δ:: NatMX4* | This work |
| 63Sc60.10 | 55Sc34.08 with *ubp10-13Myc:HphMX4 pol30-3FLAG:KanMX6 rad18Δ::NatMX4* | This work |
| 63Sc74.10 | 55Sc34.08 with *ubp10-13Myc:HphMX4 rad18-3Ha:KanMX6* | This work |
| 62Sc29.09 | 55Sc34.08 with *rev1-13Myc:HIS3* | This work |
| 62Sc30.09 | 55Sc34.08 with *rev1-13Myc:HIS3 ubp10Δ::NatMX4* | This work |
| 62Sc31.09 | 55Sc34.08 with *rev1-13Myc:HIS3 pol30-3FLAG:KanMX6* | This work |
| 62Sc32.09 | 55Sc34.08 with *rev1-13Myc:HIS3 ubp10Δ::NatMX4 pol30-3FLAG:KanMX6* | This work |
| 61Sc72.09 | 55Sc34.08 with *ubp8Δ::KanMX6* | This work |
| 61Sc73.09 | 55Sc34.08 with *ubp8Δ::KanMX6 ubp10Δ::HphMX4* | This work |
| 59Sc59.08 | 55Sc34.08 with *KanMX6*:*GAL1,10:GST:ubp3* | This work |
| 61Sc43.09 | 55Sc34.08 with *KanMX6*:*GAL1,10:ubp4* | This work |
| 61Sc44.09 | 55Sc34.08 with *KanMX6*:*GAL1,10:ubp8* | This work |
| 60Sc70.09 | 55Sc34.08 with *KanMX6:GAL1,10:GST:ubp10 bre1Δ::HphMX4* | This work |
| 60Sc69.09 | 55Sc34.08 with *KanMX6:GAL1,10:GST:ubp10^C371S^ bre1Δ::HphMX4* | This work |
| 63Sc57.08 | 55Sc34.08 with *KanMX6:GAL1,10:GST:ubp10 pol30^K164R^* | This work |
|  |  |  |

**Table S1** Gallego-Sánchez et al.,

Yeast strains used in this study. All indicated strains are isogenic with the W303 background.

| **Strain** | **Genotype** | **Origin** |
| --- | --- | --- |
| 60Sc63.09 | 55Sc34.08 with *URA3 ADE2 TRP1 HIS3* | This work |
| 60Sc64.09 | 60Sc63.09 with *ubp10Δ::KanMX6* | This work |
| 62Sc47.09 | 60Sc63.09 with *ubp10Δ::KanMX6 rev3Δ::HphMX4* | This work |
| 67Sc66.12 | 55Sc34.08 with *KanMX6:GAL1,10:UBP1* | This work |
| 67Sc68.12 | 55Sc34.08 with *KanMX6:GAL1,10:UBP2* | This work |
| 67Sc70.12 | 55Sc34.08 with *KanMX6:GAL1,10:UBP5* | This work |
| 67Sc72.12 | 55Sc34.08 with *KanMX6:GAL1,10:UBP6* | This work |
| 67Sc74.12 | 55Sc34.08 with *KanMX6:GAL1,10:UBP7* | This work |
| 67Sc76.12 | 55Sc34.08 with *KanMX6:GAL1,10:UBP9* | This work |
| 67Sc78.12 | 55Sc34.08 with *KanMX6:GAL1,10:UBP11* | This work |
| 63Sc57.12 | 55Sc34.08 with *KanMX6:GAL1,10:UBP12* | This work |
| 67Sc80.12 | 55Sc34.08 with *KanMX6:GAL1,10:UBP13* | This work |
| 68Sc02.12 | 55Sc34.08 with *KanMX6:GAL1,10:UBP14* | This work |
| 68Sc03.12 | 55Sc34.08 with *KanMX6:GAL1,10:UBP15* | This work |
| 68Sc05.12 | 55Sc34.08 with *KanMX6:GAL1,10:UBP16* | This work |
| 68Sc08.12 | 55Sc34.08 with *KanMX6:GAL1,10:UBP17* | This work |
| 69Sc47.12 | 55Sc34.08 with *rad18-3Ha:KanMX6* | This work |
| 68Sc11.12 | 55Sc34.08 with *rev3-13Myc:HphMX4* | This work |
| 68Sc18.12 | 55Sc34.08 with *rev7-13Myc:HphMX4* | This work |
| 68Sc16.12 | 55Sc34.08 with *rev3-13Myc:HphMX4 pol30-3FLAG:KanMX6* | This work |
| 68Sc17.12 | 55Sc34.08 with *rev3-13Myc:HphMX4 pol30-3FLAG:KanMX6 ubp10Δ*::*NatMX4* | This work |
| 68Sc21.12 | 55Sc34.08 with *rev7-13Myc:HphMX4 pol30-3FLAG:KanMX6* | This work |
| 68Sc24.12 | 55Sc34.08 with *rev7-13Myc:HphMX4 pol30-3FLAG:KanMX6 ubp10Δ*::*NatMX4* | This work |
| 69Sc25.12 | 55Sc34.08 with *rev1-13Myc:HphMX4 pol30^K164R^-3FLAG:KanMX6* | This work |
| 69Sc48.12 | 55Sc34.08 with *rev1-13Myc:HphMX4 pol30^K164R^-3FLAG:KanMX6 ubp10Δ*::*NatMX4* | This work |
| 61Sc08.09 | **W303** *Matα RAD5 pol30^K127R^* | T. Hishida |
| 69Sc50.12 | **W303** *Matα RAD5 pol30^K127R^ ubp10Δ*::*NatMX4* | This work |
|  |  |  |

**Table S1** Gallego-Sánchez et al.,

Yeast strains used in this study. All indicated strains are isogenic with the BY4741 background.

| **Strain** | **Genotype** | **Origin** |
| --- | --- | --- |
| 58Sc45.08 | **BY4741** *Mata his3Δ1 leu2Δ0 met15Δ0 ura3Δ0* | EUROSCARF |
| 58Sc46.08 | 58Sc45.08 with *ubp1Δ::KanMX6* | EUROSCARF |
| 58Sc47.08 | 58Sc45.08 with *ubp2Δ::KanMX6* | EUROSCARF |
| 58Sc48.08 | 58Sc45.08 with *ubp3Δ::KanMX6* | This work |
| 58Sc49.08 | 58Sc45.08 with *ubp4Δ::KanMX6* | S. Svejstrup |
| 58Sc50.08 | 58Sc45.08 with *ubp5Δ::KanMX6* | EUROSCARF |
| 58Sc51.08 | 58Sc45.08 with *ubp6Δ::KanMX6* | EUROSCARF |
| 58Sc52.08 | 58Sc45.08 with *ubp7Δ::KanMX6* | EUROSCARF |
| 58Sc53.08 | 58Sc45.08 with *ubp8Δ::KanMX6* | EUROSCARF |
| 58Sc54.08 | 58Sc45.08 with *ubp9Δ::KanMX6* | EUROSCARF |
| 58Sc55.08 | 58Sc45.08 with *ubp10Δ::KanMX6* | This work |
| 58Sc56.08 | 58Sc45.08 with *ubp11Δ::KanMX6* | EUROSCARF |
| 58Sc57.08 | 58Sc45.08 with *ubp12Δ::KanMX6* | EUROSCARF |
| 58Sc58.08 | 58Sc45.08 with *ubp13Δ::KanMX6* | EUROSCARF |
| 58Sc59.08 | 58Sc45.08 with *ubp14Δ::KanMX6* | EUROSCARF |
| 58Sc60.08 | 58Sc45.08 with *ubp15Δ::KanMX6* | EUROSCARF |
| 58Sc61.08 | 58Sc45.08 with *ubp16Δ::KanMX6* | EUROSCARF |
| 58Sc62.08 | 58Sc45.08 with *ubp17Δ::KanMX6* | EUROSCARF |
| 63Sc24.10 | 58Sc45.08 with *HIS3:GAL1,10:GST:UBP10* | This work |
| 63Sc08.10 | 58Sc45.08 with *ubp1Δ::KanMX6 HIS3:GAL1,10:GST:UBP10* | This work |
| 63Sc09.10 | 58Sc45.08 with *ubp2Δ::KanMX6 HIS3:GAL1,10:GST:UBP10* | This work |
| 63Sc10.10 | 58Sc45.08 with *ubp3Δ::KanMX6 HIS3:GAL1,10:GST:UBP10* | This work |
| 63Sc11.10 | 58Sc45.08 with *ubp4Δ::KanMX6 HIS3:GAL1,10:GST:UBP10* | This work |
| 63Sc12.10 | 58Sc45.08 with *ubp5Δ::KanMX6 HIS3:GAL1,10:GST:UBP10* | This work |
| 63Sc13.10 | 58Sc45.08 with *ubp6Δ::KanMX6 HIS3:GAL1,10:GST:UBP10* | This work |
|  |  |  |

**Table S1** Gallego-Sánchez et al.,

Yeast strains used in this study. All indicated strains are isogenic with the BY4741 background.

| **Strain** | **Genotype** | **Origin** |
| --- | --- | --- |
|  |  |  |
| 63Sc14.10 | 58Sc45.08 with *ubp7Δ::KanMX6 HIS3:GAL1,10:GST:UBP10* | This work |
| 63Sc15.10 | 58Sc45.08 with *ubp8Δ::KanMX6 HIS3:GAL1,10:GST:UBP10* | This work |
| 63Sc16.10 | 58Sc45.08 with *ubp9Δ::KanMX6 HIS3:GAL1,10:GST:UBP10* | This work |
| 63Sc17.10 | 58Sc45.08 with *ubp11Δ::KanMX6 HIS3:GAL1,10:GST:UBP10* | This work |
| 63Sc18.10 | 58Sc45.08 with *ubp12Δ::KanMX6 HIS3:GAL1,10:GST:UBP10* | This work |
| 63Sc19.10 | 58Sc45.08 with *ubp13Δ::KanMX6 HIS3:GAL1,10:GST:UBP10* | This work |
| 63Sc20.10 | 58Sc45.08 with *ubp14Δ::KanMX6 HIS3:GAL1,10:GST:UBP10* | This work |
| 63Sc21.10 | 58Sc45.08 with *ubp15Δ::KanMX6 HIS3:GAL1,10:GST:UBP10* | This work |
| 63Sc22.10 | 58Sc45.08 with *ubp16Δ::KanMX6 HIS3:GAL1,10:GST:UBP10* | This work |
| 63Sc23.10 | 58Sc45.08 with *ubp17Δ::KanMX6 HIS3:GAL1,10:GST:UBP10* | This work |
| 61Sc80.09 | 58Sc45.08 with *ubp10Δ::HphMX4* | This work |
| 61Sc81.09 | 58Sc45.08 with *rev3Δ::KanMX6* | This work |
| 62Sc01.09 | 58Sc45.08 with *ubp10Δ::HphMX4 rev3Δ::KanMX6* | This work |
| 69Sc14.12 | 58Sc45.08 with *rev3Δ::KanMX6 HIS3:GAL1,10:GST:UBP10* | This work |
|  |  |  |
